# Supplementary material for: Leaving no one behind: using action research to promote male involvement in maternal and child health in Iringa region, Tanzania
Source: BMJ Open. 2020 Nov 14;10(11):e038823. doi: 10.1136/bmjopen-2020-038823 (PMC7668372; doi:10.1136/bmjopen-2020-038823)
Supplement: Supplementary data [file bmjopen-2020-038823supp001.pdf]

Improving Access to Health Services and Quality of Care for Mothers and Children in Tanzania (IMCHA)

Women Group Meetings

By

Institute of Development Studies, University of Dar es Salaam,
Health Bridge Foundation of Canada
And
Regional Commissioner's Office (Health Department) of Iringa Region

May, 2017

Maluka S, *et al. BMJ Open* 2020; 10:e038823. doi: 10.1136/bmjopen-2020-038823

The Women Groups will complete a cycle of problem identification and prioritization, developing strategies, implementation of the strategies and evaluating the strategies to improve maternal and child health in their village. In order to complete one cycle Women Groups will conduct 8 meetings on a monthly basis.

Meeting 1: Identifying maternal and neonatal problems in the community

- Through questions and answers collect the women group's maternal and neonatal problems:
- Place all the picture cards on the floor.
- Ask each women to select one.
- Ask each women in turn, to describe the problem which she sees in the picture, what it is called (if it has a particular name), what causes this problem
- Write down the descriptions in a table on newsprint (see example in the box).
- If there are any problems other than what is shown in the picture cards, please add.

Meeting 2: Sharing the identified problem with men in the village: The objective is to make men aware of the problems and provide opportunity for men to participate in the process.

Meeting 3: Prioritizing problems - To decide on the most important problems that need to be addressed

Activities

To decide 3-5 most important maternal and neonatal health problems that need to be addressed in the community.

Discuss problems which were identified in the previous meeting

- Ask which 3-5 are the most important maternal and neonatal problems, to try to deal with in the community?
- Discuss and arrive at a consensus of the 3-5 priority problems.

Discussion points:

- What are the most frequent problems that we see on the newsprint?
- What problems are directly related to maternal and neonatal health?
- Can we deal with these problems in the community?

Meeting 4: Discuss possible strategies for addressing the priority problems

Activities

- To discuss possible strategies for addressing the priority problems.
- Remind the women of the three priority problems, which were identified in the last meeting.
- With the first problem, ask, what could we do when this problem arises?
- Discuss the different strategies for this problem. Probing maybe needed.
- List the different strategies.
- Explore, what resources the community has to deal with this problem?
-

Possible Resources

Who	What can they do
Women's group	▪ Create awareness<br>▪ Create pressure<br>▪ Provide local equipment/labour

	▪ Plan, implement, monitor and evaluate the activities
IMCHA project	▪ Facilitate to create awareness<br>▪ Facilitate to mobilise group<br>▪ Co-ordinate and liaise with other organisations<br>▪ Strengthen the health institutions & committees
Community	▪ Participate in the different strategies<br>▪ Provide suggestions and feedback<br>▪ Provide public property, such as building, furniture, equipment, utensils<br>▪ Demand and create pressure
Health Facility	▪ Provide health service and treatment<br>▪ Provide medicine & medical supplies<br>▪ Run outreach and immunisation clinic<br>▪ Create awareness
Other organizations	▪ Provide medicines and equipment<br>▪ Organise health camp and clinics<br>▪ Support economically<br>▪ Provide training

- Discuss, and list the strategies and the resources and make realistic strategies.
- Repeat these steps for the second and third priority problems.

Meeting 5: Preparing for the community members meeting

Activities
To prepare for the community members meeting.

- **Discussion points**:
  - ✓ When will the community meeting take place?
  - ✓ Where will it take place?
  - ✓ Who will invite the participants?
  - ✓ How will they be invited? (verbally, by letter)
  - ✓ Who will be responsible for this?
- Discuss about preparing the verbal presentations. **Discussion points**:
  - ✓ Who can explain what the group did, at the community meeting?
  - ✓ Who can explain about the three problems, at the community meeting?
  - ✓ Who can explain about the strategies, at the community meeting?
  - ✓ What is the best way of presenting this information? (charts, drawings)
  - ✓ Should one woman explain all or should different women explain different things?
- Discuss about preparing the socio-drama
  - ✓ What should the drama be about?
  - ✓ Who will write the story?
  - ✓ Who will perform as actors?

Meeting 6: Presenting to the community meeting

Activities

- ***The community members will learn about what the women have been doing*** - The WGF needs to open the meeting, briefly introduce IMCHA project, and introduce the women, their work and the purpose of this meeting to the community members.
- ***The community members will learn about the three problems identified by the women-*** The women will present how the information on the problems was collected. The women need to present their three priority problems (verbal presentations OR

socio-drama OR both).

- ***The community members will learn about the possible strategies that the women have suggested to address the three problems*** - The women need to present their three problems and the possible strategies to address these problems.
- to reach a consensus of the strategies

Meeting 7: Refining and Implementation of the Strategies

- **Agree on the strategies-** The WGFs needs to show the table of strategies on newsprint (such as the one in the box below).
- Add any strategies that have been developed from the meeting and remove any strategies that are not considered suitable.
- Reach a consensus on the strategies.
- Develop a plan for each strategy. When will the strategy be implemented? Who will be responsible for each strategy?

Examples of implementation plan

Possible problem	Realistic Strategies	When the strategy will be implemented	Person responsible
Home delivery	Women's group teaches other women about the danger of home delivery	June 2017	Women Group Members
	Women's group organizes street drama	July 2017	Women Group Members
	Women groups and male champions visit couples at home to discuss birth plans	September 2017	Women Group Members and Male champions
Early ANC attendance	Women's group teaches other women about the importance of early ANC attendance	June 2017	Women Group Members
	Male champions teaches other men the importance of early ANC attendance	July 2017	Men Group Members
Home delivery	Women's group teaches other women about the danger of home delivery	June 2017	Women Group Members
	Women's group organizes street drama	July 2017	Women Group Members

Meeting 8: Evaluating together

The Women's Group Facilitators will guide the women and communities in their area through a process of evaluating the strategies they have implemented and planning for the future in terms of improving the strategies and identifying new problems and how to address them.

Maluka S, *et al. BMJ Open* 2020; 10:e038823. doi: 10.1136/bmjopen-2020-038823
