# Supplementary material for: Leaving no one behind: using action research to promote male involvement in maternal and child health in Iringa region, Tanzania
Source: BMJ Open. 2020 Nov 14;10(11):e038823. doi: 10.1136/bmjopen-2020-038823 (PMC7668372; doi:10.1136/bmjopen-2020-038823)
Supplement: Author's manuscript [file bmjopen-2020-038823.draft_revisions.pdf]

BMJ Open is committed to open peer review. As part of this commitment we make the peer review history of every article we publish publicly available.

When an article is published we post the peer reviewers' comments and the authors' responses online. We also post the versions of the paper that were used during peer review. These are the versions that the peer review comments apply to.

The versions of the paper that follow are the versions that were submitted during the peer review process. They are not the versions of record or the final published versions. They should not be cited or distributed as the published version of this manuscript.

BMJ Open is an open access journal and the full, final, typeset and author-corrected version of record of the manuscript is available on our site with no access controls, subscription charges or pay-per-view fees (http://bmjopen.bmj.com).

If you have any questions on BMJ Open's open peer review process please email info.bmjopen@bmj.com

BMJ Open

Leaving no one behind: using action research to promote male involvement in maternal and child health in Iringa region, Tanzania

Journal:	*BMJ Open*
Manuscript ID	bmjopen-2020-038823
Article Type:	Original research
Date Submitted by the Author:	25-Mar-2020
Complete List of Authors:	Maluka, Stephen; University of Dar es Salaam, Institute of Development Studies<br>Japhet, Paul; University of Dar es Salaam, Institute of Development Studies<br>Fitzgerald, Sian; Healthbridge Foundation<br>Begum, Khadija; Health bridge Foundation<br>Alexander, May; Iringa Region, Health Department<br>Kamuzora, Peter; University of Dar Es Salaam, Institute of Development Studies
Keywords:	Antenatal < GENETICS, PUBLIC HEALTH, Maternal medicine < OBSTETRICS

SCHOLARONE™
Manuscripts

I, the Submitting Author has the right to grant and does grant on behalf of all authors of the Work (as defined in the below author licence), an exclusive licence and/or a non-exclusive licence for contributions from authors who are: i) UK Crown employees; ii) where BMJ has agreed a CC-BY licence shall apply, and/or iii) in accordance with the terms applicable for US Federal Government officers or employees acting as part of their official duties; on a worldwide, perpetual, irrevocable, royalty-free basis to BMJ Publishing Group Ltd ("BMJ") its licensees and where the relevant Journal is co-owned by BMJ to the co-owners of the Journal, to publish the Work in this journal and any other BMJ products and to exploit all rights, as set out in our licence.

The Submitting Author accepts and understands that any supply made under these terms is made by BMJ to the Submitting Author unless you are acting as an employee on behalf of your employer or a postgraduate student of an affiliated institution which is paying any applicable article publishing charge ("APC") for Open Access articles. Where the Submitting Author wishes to make the Work available on an Open Access basis (and intends to pay the relevant APC), the terms of reuse of such Open Access shall be governed by a Creative Commons licence – details of these licences and which Creative Commons licence will apply to this Work are set out in our licence referred to above.

Other than as permitted in any relevant BMJ Author's Self Archiving Policies, I confirm this Work has not been accepted for publication elsewhere, is not being considered for publication elsewhere and does not duplicate material already published. I confirm all authors consent to publication of this Work and authorise the granting of this licence.

For peer review only

Leaving no one behind: using action research to promote male involvement in maternal and child health in Iringa region, Tanzania

Stephen Maluka[1*], Paul Japhet [2], Sian Fitzgerald[3], Khadija Begum[3], May Alexander[4] & Peter Kamuzora[2]

1. University of Dar es Salaam College of Education (DUCE), P.O.BOX 2329, Dar es Salaam, Tanzania
2. Institute of Development Studies, University of Dar es Salaam. P.O.BOX 35169, Dar es Salaam, Tanzania
3. Health bridge Foundation of Canada.
4. Iringa Municipal Council, Health Department. Iringa, Tanzania

SOM: stephenmaluka@yahoo.co.uk
JP: pijsamba@yahoo.com
SF: sfitzgerald@healthbridge.ca
KB: khadijabgm@gmail.com
MA: may20tz@yahoo.com
PK: petkamu@Udsm.ac.tz

* Corresponding Author
SOM: stephenmaluka@yahoo.co.uk

For peer review only

ABSTRACT

Introduction

Male involvement has been reported to improve maternal and child health (MCH) outcomes. However, most studies in Low and Middle Income Countries (LMIC) have reported low participation of men in MCH related programmes. While there is a growing interest in the involvement of men in maternal and child health, little is known on how male involvement can be effectively promoted in settings where entrenched unequal gender roles, norms and relations constrain women from effectively inviting men to participate in maternal and child health.

Methods and analysis

This paper reports participatory action research aimed to promote male participation in pregnancy and childbirth in Iringa Region, Tanzania. As part of the Innovating for Maternal and Child Health in Africa (IMCHA) project, participatory Action Research (PAR) was conducted in 20 villages in two rural districts in Tanzania. Men and women were engaged separately to identify barriers to male involvement in antenatal care (ANC) and during delivery; and then they were facilitated to design strategies to promote male participation in their communities. Along with the PAR intervention, researchers undertook a series of research activities. A thematic analysis was used to analyse the data. The common strategies designed were: engaging health facility committees; using male champions and male gatekeepers; and using female champions to sensitise and provide health education to women. These strategies were validated during stakeholders' meetings, which were convened in each community.

Discussion

The use of participatory approach not only empowers communities to diagnose barriers to male involvement and develop culturally acceptable strategies but also increases sustainability of the interventions beyond the life span of the project. More lessons will be identified during the implementation of these strategies.

Key Words: Male involvement; Maternal and Child Health; Participatory Action Research; Tanzania

For peer review only - http://bmjopen.bmj.com/site/about/guidelines.xhtml

ARTICLE SUMMARY

Article Focus

- While there is a growing interest in the involvement of men in maternal and child health, little is known on how male involvement can be effectively promoted in settings where entrenched unequal gender roles, norms and relations constrain women from effectively inviting men to participate in maternal and child health.
- This paper makes a valuable contribution to the literature on male involvement in maternal and child health because it addresses the question of how male involvement can be promoted.

Key Messages

- The use of participatory approach not only empowers communities to diagnose barriers to male involvement and develop culturally acceptable strategies but also increases sustainability of the interventions beyond the life span of the project.

Strengths and Limitations

- While action research has been used extensively in the health sector, there has been little evidence of its relevance to promoting male involvement in maternal and child health. This study is one of the few to have used action research approach to promote male involvement in maternal and child health.
- The findings reported are based on the initial stages of the participatory action research. The programme is now going into the fourth and fifth phases of implementation and evaluation of the effects of the strategies on male involvement in maternal and child health.

For peer review only

1. Introduction

Maternal and Child death is a global problem and is highly overwhelming in low and middle income countries. An estimate of 303,000 pregnant women and 8.4 million under-five children died in 2015 [1]. Low and middle-income countries account for approximately 99% of all deaths [2-3]. In Tanzania, the maternal mortality rate is 556 per 100,000 live births while the infant and under-five mortality rates are 43/1,000 and 67/1,000 respectively [4]. The neonatal mortality rate is 25 per 1,000 live births [4].

Low utilisation of maternal and child health services is mentioned as one of the root causes of maternal and child deaths [1-3]. In Tanzania, for example, though over 90% of pregnant women attend antenatal care (ANC) at least once, only 51% complete four or more recommended visits, whereas only 24% of women attend their first ANC before the fourth month of pregnancy [4-5]. Health facility deliveries were 63% while deliveries assisted by health professional were 64% [4]. Furthermore, home deliveries and deliveries without a help of health professionals were 37% and 36% respectively; and only 13% of women who delivered at home attended postnatal care (PNC) within the recommended timeline of 48 hours after delivery [4].

In Tanzania, like in other African countries, the adverse effect of socio-economic factors on maternal, new-born and child health is compounded by gender inequalities that limit women's autonomy and decision-making power [4]. This prevents women and their families from practicing healthy behaviours as well as seeking and receiving life-saving care at and around the time of birth [4, 6-9]. The delay in seeking care and delay in reaching healthcare facilities occurs when women lack support from male partners, especially when the situation involves the need for money [9-10].

Male involvement has been reported to improve maternal and child health outcomes. Among the perceived benefits associated with increased male involvement include increased access to ANC and delivery at the health facility with assistance of skilled birth attendants [11]; increased knowledge of both women and men on the obstetric care emergencies [12-13]; increased use of family planning methods [14-15]; and decreased gender-related barriers to maternal and child health services [16-17].

Globally, a number of strategies have been implemented to increase male involvement in maternal and child health services. The strategies include health education campaigns on the mass media, at work places, community outreach, and counselling of couples at health facilities [18-19]. Others are fast tracking to couples who attend clinics together [18-22], denying services to women attending ANC without male partners and use of by-laws [19-23]. However, there is limited evidence for the relative effectiveness of various strategies to promote male involvement.

In Tanzania, policies, guidelines and strategies emphasize male involvement in maternal and child health services. The focused antenatal care (FANC) guidelines require pregnant women to attend clinics with their partners as part of the prevention of mother to child transmission (PMTCT). Pregnant women are, therefore, required to attend the first ANC visits with their male partners for HIV testing [24-26]. Despite these strategies, only 30% of male partners participate in the couple counselling and HIV testing [26].

While there is a growing interest in the involvement of men in maternal and child health, little is known on how male involvement can be effectively promoted in settings where entrenched

For peer review only - http://bmjopen.bmj.com/site/about/guidelines.xhtml

unequal gender roles, norms and relations constrain women from effectively inviting men to participate in maternal and child health. Most policies take a narrow definition of male involvement focusing more on clinic attendance. In this study, male involvement refers to provision of social, informational and psychological support to pregnant women. This paper makes a valuable contribution to the literature on male involvement in maternal and child health because it addresses the question of how male involvement can be promoted.

2. Methods

2.1 Study Settings and Design

This study was part of the large project under the Innovating for Maternal and Child Health in Africa (IMCHA) programme, which is being implemented in Kilolo and Mufindi districts in Iringa region, Tanzania. IMCHA programme (September 2015- March 2020) aimed to improve maternal, new-born and child health outcomes by strengthening health systems to become more equitable, using primary health care as an entry point. Iringa region was selected because of the earlier research collaboration between some of the IMCHA researchers and the regional and district decision makers on strengthening decentralised district health management. The Districts are predominantly rural and the majority of the population rely on agriculture for subsistence and production.

A participatory action research (PAR) design was adopted in this study [31]. Action research is the research conducted in partnership with members of the community mainly aimed at bringing about structural or cultural change [31-32]. The PAR process consisted of a series of meetings during which women, men and community stakeholders identified barriers to male involvement in maternal and child health; and then designed strategies to promote male participation. The Action Research Team (ART) led by the researchers from the University of Dar es Salaam, in collaboration with the regional and district health managers from Iringa Region facilitated the PAR. As indicated in Figure 1, the PAR process involved five phases.

Figure 1: Participatory Action Research Cycle

2.1.1 Introduction and Formation of Women and Men Groups

We conducted a series of consultation meetings with the community members in 20 intervention villages. First, the ART introduced the project to the community and facilitated the formation of women and men groups in 20 intervention villages. Women and men groups comprised 20 and 10 members respectively, who were selected during community meetings facilitated by the ART in each intervention village. The criteria for selecting women included: age 15-49 years, experience in utilizing MCH services, and women who were pregnant or had given birth in the last 12 months preceding the study. The criteria for selecting male participants included: having a wife/female partner and experience with childbirth. In total, 400 women and 200 men were identified and included in the groups. In addition, 40 community health workers (CHWs); two (2) from each village were identified and included in the women groups. Women groups together with CHWs were given training on how they could diagnose various barriers to the access and use of maternal and child health services in their community; and then designed strategies to address these barriers.

For peer review only - http://bmjopen.bmj.com/site/about/guidelines.xhtml

2.1.2 Diagnosing and prioritizing problems

In Phase II, the ART led the women groups in identifying and prioritising barriers to male involvement in maternal and child health in their community. Women were able to identify barriers without the presence of men. In the next meeting, the ART facilitated women groups to share and discuss the identified barriers with male champions in their community. The rationale of including men in the meetings was to give them an opportunity to share their views as key stakeholders and decision makers in maternal and child health matters. This process also aimed to prepare men to actively participate in the design and implementation of the strategies.

2.1.3 Designing male engagement strategies

In Phase III, women and men together developed strategies to promote male involvement in maternal and child health. In addition, the ART facilitated community meetings where women groups and male champions shared the barriers and strategies designed to promote male involvement. The key stakeholders who participated in the community meetings included the government and community leaders, religious leaders, health providers and members of the user committees. The objective of the community meetings was to give stakeholders an opportunity to discuss and refine the problems, root causes and strategies identified by women groups. In addition, the community meetings aimed at actively involving community stakeholders in the implementation of the strategies in their respective areas. As indicated in Table 1, an average of 45 stakeholders participated in each community meeting.

Table 1: Participants by type attending community meetings

**Districts**	**Kilolo**					**Mufindi**					**Total**
**Wards**	1	2	3	4	5	6	7	8	9	10	
District Representatives	1	1	1	1	1	-	-	-	-	-	**5**
Ward Councillors	1	1	1	1	1	1	1	1	-	1	**9**
Ward Executive Officers	1	1	1	1	1	1	1	1	1	1	**10**
Community Development Officers	1	-	1	-	1	-	-	-	-	1	**4**
Village Executive Officers	2	2	2	1	1	2	2	2	2	2	**18**
Village Chair persons	2	2	2	2	2	2	2	2	2	1	**19**
Religious Leaders	3	5	2	4	4	3	3	4	6	3	**37**
Hamlet Chairs	9	10	10	14	9	10	10	10	11	8	**101**
Primary School Representatives	2	1	1	1	1	2	2	2	3	2	**17**
Secondary School Representatives	1	1	-	1	1	-	-	-	1	-	**5**
Health Facility Staff (Clinician)	1	1	1	1	1	2	1	-	-	1	**9**
Health Facility Governing Committees	1	1	1	1	-	1	1	1	-	1	**8**
Community Health Workers	4	4	4	4	4	3	3	4	4	4	**38**
Male Champions	6	6	6	7	6	5	2	6	3	3	**50**
Women Groups Representatives	10	13	10	10	10	10	8	12	10	11	**104**
Others	3	2	2	2	2	1	3	1	-	-	**16**
Total	**48**	**51**	**45**	**51**	**45**	**43**	**39**	**46**	**43**	**39**	**450**

2.1.4 Implementing and evaluating strategies

In Phase IV, women and men groups developed plans and led the implementation of the strategies to promote male involvement in maternal and child health. In Phase V, women groups and male champions together evaluated the effectiveness of the strategies. In this paper, we report the first three phases of the PAR as the last two phases (implementation and evaluation) were not yet completed. As indicated in Table 2, in total, we facilitated 102 meetings between February 2017 and March 2018 for the first three phases.

Table 2: Meetings facilitated by the ART in the communities

S/N	*Type of meeting*	*Number*
1	Orientation and formation of women and men groups	20
2	Training of women group members	10
3	Training of Community Health Workers	2
4	Identification and prioritisation of barriers to MCH, including male involvement	20
5	Sharing identified barriers with men and jointly designing strategies	20
6	Preparing women group members for the community meetings	20
7	Community/stakeholders meetings to discuss and refine barriers, root causes and strategies	10
	**Total meetings**	**102**

2.2 Data collection techniques

As part of the PAR intervention, the researchers conducted a series of research activities. All the meetings conducted with women, men and community members were documented. In some cases, the discussions were audio-recorded with permission from the participants. Where recording was not possible, notes from the discussion were taken. In addition, trained research assistants prepared reports for the meetings. Similarly, all the presentations made by the women groups during community meetings were audio-recorded. Furthermore, a structured questionnaire was used to collect data on socio-demographic characteristics, men's knowledge and awareness of family planning and ANC services and their practices in relation to prenatal care and childbirth, and related decision making power. Data were collected through digital tablets using Open Data Kit (ODK) software by five local interviewers between June and July 2018.

2.3 Data analysis

All recordings from the women, men and community meetings were transcribed verbatim by trained transcribers. The transcripts were reviewed by the core research team members and notes were made for each transcript. The transcripts and summaries of the stakeholders' meetings were analysed using a thematic approach [33]. First, the Principal Investigator (SOM) developed a code manual based on the objectives of the study. The coding framework was further discussed and refined by the research team members. PJ and SOM manually coded the transcripts and summaries of the meetings to the identified codes. Additional codes, which emerged during coding process, were added along the way. PK cross-checked the coding process. Responses were then compared across different categories of participants. Finally, data were summarized and synthesized, keeping the key expressions of respondents as illustrative cases. Quantitative data analyses were performed using Stata version 15.0 (Stata Corporation, College Station, TX, USA).

2.4 Patient and Public Involvement

This study was conducted in partnership with members of the community. The participatory

For peer review only - http://bmjopen.bmj.com/site/about/guidelines.xhtml

action research process consisted of a series of meetings during which women, men and community stakeholders identified barriers to male involvement in maternal and child health and then designed strategies to promote male participation. Findings were shared during stakeholders meetings held in the respective communities.

3. Findings

This section presents the key findings of the study. The key themes covered are: characteristics of women and men groups, barriers to male involvement in maternal and child health and strategies designed to promote male involvement.

3.1.1 Characteristics of the women group members

The mean age (±SD) of the women respondents was 35.7±9.4 (Median=35, Min=16 and Max=61). Most of the women (75%) were between 26 and 49 years of age. About 78% (n=208) of the women were married, and most of them were living with their husbands (96%). Among the married women, 21% (n=44) reported that their husbands had more than one wife. Table 3 shows the main characteristics of the women group members.

Table 3: Characteristics of the women

Characteristics	N	%
**Age category**		
16 to 25 y	41	15.5
26 to 35 y	98	37.0
36 to 49 y	100	37.7
50 to 65 y	26	9.8
**Marital status**		
Never married	29	10.9
Married	208	78.5
Divorced/separated	3	1.1
Widows	25	9.4
**Highest level of education**		
Never attended school	6	2.3
Primary, not completed	15	5.7
Primary, completed	191	72.1
Secondary, not completed	22	8.3
Secondary, completed	28	10.6
Vocational/ adult education	3	1.1
**Primary occupation of the women**		
Farmers	227	85.7
Non-farmers	38	14.3
**Primary occupation of husbands**		
Farmers	146	73.0
Non-farmers	54	27.0

In terms of antenatal care and childbirth, around 9% (23 of 265) of the women group members were pregnant during the interviews, and 19 (83%) of them reported to attend ANC. We also explored prenatal and childbirth care practices of these women during their immediate pregnancy. Although around 96% of the women (254 of 265) attended antenatal care at health facilities, only about 72% of them (184 of 254) had had four or more ANC visits, and 93% (235 of 254) gave birth at health facilities. The prevalence of ≥4 ANC visits

For peer review only - http://bmjopen.bmj.com/site/about/guidelines.xhtml

and facility births were low among all the women interviewed - 69% and 91% respectively (Figure 2). Figure 2 also shows that although 93% of the women (235 of 254) reported receiving iron-folic acid (IFA) tablets from health facilities, adherence to recommended consumption was much low (37%).

Figure 2: Prevalence of prenatal and delivery care among women group members

3.1.2 Characteristics of male champions

As indicated in Table 5, about 51% (90) of the male champions were between the age range of 20-39. Moreover, 82% (144) had completed primary education and nearly all (98%) were married. As for women group members, the majority (92%) of the male champions relied on agriculture for subsistence and production.

Table 5: Male demographic characteristics

Male demographic characteristics	n	%
Age Range		
20-39	90	51
40-59	79	45
Above 60	6	3
Level of Education		
Not completed Primary school	2	1
Completed Primary school	144	82
Not completed Secondary School	11	6
Completed secondary School	15	9
College/University	3	2
Marital status		
Single	2	1
Married	172	98
Widower	1	1
Occupation		
Employed	2	1
Farmer	161	92
Self-employed/small business	12	7
Number of children		
1-3	85	49
4-6	69	39
7+	21	12

We also assessed the awareness and knowledge of male champions on various maternal and child health matters. As indicated in Table 6, nearly all participants (99%) were aware of the family planning (FP). However, most men (65%) only mentioned condom as the main FP method. Other methods of FP planning were rarely mentioned. The majority of the participants (84%) understood the role of ANC as taking care of pregnant women and their foetus. However, only 21% (63 of 175) agreed that men were required to accompany their

For peer review only - http://bmjopen.bmj.com/site/about/guidelines.xhtml

wives for ANC visits. Furthermore, 80% (125 of 156) had knowledge of exclusive breastfeeding (EBF) and 78% (136 of 175) had knowledge of the duration of the EBF.

Table 6: Men's awareness and knowledge of maternal and child health matters

Awareness and knowledge of male champions on MCH	n	%
Ever heard of family planning		
Yes	173	99
No	1	1
Meaning of family planning		
Control of family size	62	26
Child spacing	145	61
Preventing unwanted pregnancy	32	13
Men's role in family planning		
Consent	54	27
Support	145	73
Others (Specify)	0	0
I don't know	0	0
Awareness of contraceptive methods for men		
Vasectomy	10	5
Male condom	144	65
Injectables	32	14
Diaphram	4	2
Hormonal contraception, such as the pill	32	14
What ANC entails		
Taking care of pregnant women and their foetus	162	84
Giving drugs and injection to pregnant women	9	5
Detecting and managing complications	21	11
Men's role in ANC		
Financial support	24	8
Encouraging and reminding pregnant women	105	36
Providing emotional and moral support	103	35
Accompanying pregnant women to clinic visits	63	21
Knowledge of exclusive breastfeeding (EBF)		
Breast milk alone	125	80
Breast milk with little water	3	2
No knowledge	28	18
Knowledge of the duration of EBF		
Below 6 months	13	7
6 months	136	78
Above 6 months	10	6
No knowledge	16	9

3.2 Barriers to male involvement

While majority of women preferred to attend first ANC appointment with their partners, men did not wish to be actively involved in ANC and delivery. Participants perceived men as

For peer review only - http://bmjopen.bmj.com/site/about/guidelines.xhtml

breadwinners and their main role in pregnancy and child birth was to support their partners financially. There were contradicting views regarding the need for men to assist their wives with household chores; while women preferred to be assisted with household chores during pregnancy and early days after birth, majority of male participants felt that this was mainly women's responsibility. Furthermore, it was evident that men did not prefer their wives to use modern family planning methods due to fear of side effects. Men also had the perception that the use of family planning would increase cheating for married women. Table 7 provides a summary of the barriers to male involvement as identified in most of the intervention villages.

Table 7: Barriers to male involvement in maternal and child health

Problem	Root Causes
Low male involvement in maternal and child health	Lack of health education, especially for men. Most often health education is provided by health workers during ANC clinics. Men rarely attended ANC clinics
	Traditional gender roles and norms. Maternal and child health is considered as women's affair. Men's role is mainly supporting women financially
	Fear of HIV testing. As part of the PMTCT programme, couples are required to test for HIV during ANC appointment. Men always prefer their wives to test first and assume that the wives' results would be the same to the husband.
	Low birth spacing makes men fear to accompany their wives. Men think that they would be reprimanded by health providers and fellow men
	Couple relationships may encourage or hinder male participation in maternal and child health matters. The better the relationship, the higher the male participation and vice versa.
	Unfavourable environment in the health facilities. Most mentioned environments including long waiting time, disrespectful languages of the health providers, and lack of physical space to accommodate men and women concurrently.

3.3 Strategies designed to promote male involvement

The key strategies which were commonly proposed were: engaging health facility governing committees; use of male champions and gatekeepers; and using women champions.

3.3.1 Engaging health facility governing committees

In all public primary health care facilities, there are health facility governing committees. The committees are composed of representatives of the community and health facility. The health facility governing committees have the mandate of overseeing the delivery of quality health care services and mobilizing the community to finance and manage health care services. The use of health facility governing committees was identified as a potential strategy to promote male engagement in maternal and child health services. Participants reported that actively engaging health facility governing committees would help address health facility related barriers to male involvement including long waiting time, disrespectful languages of the health providers, and lack of physical space to accommodate men and women concurrently as exemplified by one of the stakeholders:

> *The health governing committee should be coached on their roles and responsibilities to ensure they properly supervise provision of services at the health facility. If they are engaged, the committee will be able to talk to the health care*

For peer review only - http://bmjopen.bmj.com/site/about/guidelines.xhtml

workers not to harass pregnant women and their partners. The committee can also tackle other supply side related problems which hinder use of health care services. This will attract men to attend clinics (Male participant during stakeholder meeting).

3.3.2 Engaging male champions and gate keepers

There was consensus among participants that men do not accompany their wives to ANC clinics; and fear to use family planning methods because they lack education. While women most often get health education in health facilities, particularly during ANC visits, men rarely receive health education. In addition, traditional gender roles and norms hinder men's participation in maternal and child health matters. Men's perception of superiority makes it difficult for wives to pass the message from health providers to their husbands. Most often wives fear to discuss family planning, pregnancy, and child birth related matters with their husbands due to culturally entrenched power differences between wives and husbands. In this context, male champions are better positioned to educate their fellow men and ultimately break the entrenched cultural gender roles and norms. Participants suggested that male champions should be identified in each village. They should also be facilitated in terms of key messages to be delivered to men. Male champions should deliver key messages through bars, football matches and other social gatherings in the villages as explained by some participants:

Men have inherited the beliefs that they are superior to women. Women are voiceless in making and influencing decisions at the household. Even the young generation has inherited the same cultural beliefs. It is difficult for women to educate or communicate to men the information they receive from the health facilities. We need to identify male champions who will talk to their fellow men in their social gatherings (Religious leader).

Other participants added:

Most of the maternal and child health programmes are directed to women. Men do not get opportunities to receive health information becuaes they rarely attend clinics. Unfortunately, when women get the knowledge, they cannot deliver to their partners. They always fear to talk to their husbands (Female participant).

We request IMCHA project team to focus mostly on men so that we can get first-hand information instead of waiting for female partners. We have always been excluded when it comes to maternal and child health education. Women are given education in health facilities. Men rarely get these opportunities (Male participant).

Closely connected to the above, participants in various meetings underlined the importance of engaging male gate keepers particularly religious and community leaders. It was highlighted that religious and community leaders are highly respected in the community. They have the power to convey health information to men and women alike. According to our participants, male gatekeepers should be actively engaged in the sensitization campaigns or in providing education to men and women in their communities as explained by some respondents:

*We should think very carefully about people who will deliver messages or education to the community. Others are an obstacle and using them as champions will end up losing instead of gaining. People will not listen; instead they will start whispering. But if we use influential people like religious leaders, elderly, or government officials, we might be able to increase the acceptability of the messages (*Community leader).

For peer review only - http://bmjopen.bmj.com/site/about/guidelines.xhtml

Another respondent added:

> *Women have presented the truth. The challenge is to get the right people who will deliver the key messages to the community and men in particular. I think if we work with religious and community leaders, the messages can easily be accepted by men* (Male participant).

Almost all participants requested the IMCHA project team to provide training to male champions and gatekeepers. They also requested to provide them with key messages that should be delivered to the community, particularly to men. The key messages should focus on the importance of men to attend ANC clinics along with their partners, birth preparedness including shared decision making during pregnancy and child birth and the importance of family planning use.

> *This sensitization and training should not stop today. Women have presented very important issues which need to be addressed in our communities. We request researchers to provide more training to men, religious, and community leaders so that they can deliver these messages to the community. We have various platforms in our communities which can be used to communicate this information* (Government leader).

3.3.3 Women champions and education of fellow women

There was consensus among participants that relationships among couples may encourage or hinder male participation in maternal and child health matters; on the one hand, unfriendly relationship will hinder active participation of men. On the other hand, good couple relationship will facilitate male involvement in maternal and child health. In this context, the use of women groups was considered important, particularly to provide education to their fellow women on the best practices. It was reported that women have many social platforms where they could share and discuss the information related to couple relationships during pregnancy and child birth. Improved couple relationships will increase male participation in maternal and child health as elaborated by some participants:

> *Men are like children. When you need to talk to them, you should read their mind and feelings. If they are in bad mood don't say any thing to them, wait until they feel better. We need to understand the moods of our husbands and the best occasion that men can listen and understand our needs* (Female participant).

Other participants added that:

> *We, women, have problems. When a man agrees to do some household chores, we still want to make it public. We speak to our friends, neighbours or in saloons that, you just make my hair slowly, no need for hurry; my husband is taking care of children…. Even food, I will find it already cooked. If men know this, they will never support you again* (Female participant).
>
> *The way a wife communicates information to the husband may result in participation or non participation in antenatal care clinics. Some women think that when they are pregnant it is the time to harass their husbands. Men need soft language, if you use harsh or rude language, we interpret it as a command. Usually, men need respect and love. If you show us respect and love, we are ready to accompany you to ANC clinics and assist in domestic chores* (Male participant).

Another participant had this to say:

> *Women are the root cause of the low male involvement because they do not communicate with their husbands in a polite language. For example, when I come back home and tell my wife, I need the night meal (to have sex) and the wife replies*

For peer review only - http://bmjopen.bmj.com/site/about/guidelines.xhtml

rudely that 'I'm not ready', I will keep quiet. If I get the same answer the following day....I may start thinking of alternatives, looking for other women to satisfy my sexual desire. So, women need to use soft language, and we can understand them and plan together (Male participant).

2. Discussion

This study adds new knowledge on the strategies for promoting male involvement in maternal and child health in low-income and middle-income countries. It is one of the few to have used action research approach to promote male involvement in maternal and child health. While action research has been used extensively in the health sector in Tanzania [34-36] and in other low-income and middle-income countries [37- 40], there has been little evidence of its appropriateness in promoting male involvement in maternal and child health. We have demonstrated how stakeholders, including men, were able to design culturally acceptable strategies to increase male involvement in maternal and child health. The next section discusses the strategies suggested by the participants in the current study in the light of other studies in low-income and middle-income countries.

4.1 Health education and community sensitization

Health education and community sensitisation campaigns were widely suggested in many villages as an important strategy which can be used to promote male participation in maternal and child health. Participants felt that majority of community members, particularly men, lack maternal and child health education. While male champions who participated in the study had adequate knowledge of ANC care, family planning and exclusive breast feeding, this was only a small segment of the population. In addition, male champions were selected based on their interests in maternal and child health matters. It may be possible that most of the male champions had experience in maternal and child health matters. Health education interventions tailored to women and men have been effective in increasing knowledge, health seeking behaviour and raising awareness on issues related to maternal and child health [18, 41-43]. In our study, participants said that in order to make it sustainable, health education should be provided by health care workers and community health workers who already exist in the communities. They also preferred the campaigns to be integrated in the community and political gatherings in their respective villages. Participants preferred that key messages be developed to facilitate the campaigns and sensitisation programmes.

Connected to the above, participants suggested the use of male champions and male gatekeepers to promote behaviour change among men. It was evident from the findings that while women had access to health education during ANC clinics, men rarely attend health facilities. However, due to entrenched gender power relations, most often women are not able to convey the health information received from health facilities to their husbands. A study in Tanzania showed that the traditional way of using women to convey health information to male partners was a barrier to male involvement because men preferred to receive the message through other men [44]. The use of male champions is an effective strategy to include and engage men by targeting men focused key messages on the importance of participating in maternal and child health. A study in the Philippines reported that using male peer educators improved participation of men in maternal and child health matters [45]. Similarly, recent studies in Malawi underscored the importance of male champions in facilitating men's participation in maternal and child health and challenging traditional gender norms [19, 46]. Men were targeted with messages focused on the benefits of utilising maternal and child health services [19,46]. However, while in the Malawian studies male champions delivered the key messages to men secretly [19], in our study, the participants

For peer review only - http://bmjopen.bmj.com/site/about/guidelines.xhtml

suggested that male champions should discuss maternal and child health openly in the men's platforms including football matches, bars and other places where men meet. Furthermore, sensitization and support should also be provided to religious, traditional, and government leaders to spread these messages in various religious, political, and social gatherings. Studies in other Africa countries have highlighted the importance of engaging religious and traditional leaders in promoting male involvement in maternal and child health [18 -19,46].

Furthermore, participants underlined the importance of managing couple relationships in order to increase men's participation in MCH services. Female and male participants suggested that women should learn how to strategically manage their couple relationships in order not to deter male men from engaging in MCH. It is absolutely the case that men's ability and willingness to be involved in MCH is influenced by couple relationship dynamics [19,46]. However, in our study, it seems that participants recommended that the burden of emotional labour involved in managing couple relationships should fall exclusively to women, and not to men. For example, it is the women and not men who are responsible for communicating with "soft language". While this finding is an important contribution to the literature, it implies the dominance of unequal expectations of men and women in the society. Managing couple relationships should be the responsibility of both women and men.

4.2 Use of health facility governing committees

In our study, participants suggested the use of health facility governing committees to address facility related barriers to male involvement in maternal and child health. Health facility governing committees have been reported to significantly facilitate access to maternal and child health services [47]. However, in Tanzania, studies have indicated that while health facility committees exist in all public primary health care facilities, limited financial resources, limited capacity and skills, attitudes of health workers and lack of clarity of the role and mandate of committees affect the performance of the committees [48-50]. In order to maximise their performance, deliberate efforts should be made to strengthen the capacity of the health committees. Furthermore, the Ministry of Health should ensure that district Councils allocate adequate funds to the district annually to support meetings of the health committees as it is done for the council Health Services Boards (CHSBs). Studies have reported that CHSBs in Tanzania managed to attract members with good qualification mainly due to the availability of good incentives [49-50].

Limitations of the study

The main limitation of this study is that it does not report the implementation and effects of the designed strategies. The findings reported are based on the initial stages of the participatory action research. The programme is now going into the fourth and fifth phases of implementation and evaluation of the effects of the strategies on male involvement in maternal and child health. Another limitation of the study is lack of independence of the data collection. The data collection was carried out by the researchers who were part of the ART. However, the data collectors had no motivation to consciously or subconsciously skew the responses in a particular direction, although errors in data collection will always be expected. The data collectors were paid to collect the data as accurately as possible and their compensation did not depend on what data they collected. Moreover, the interviewers did not collect data from their family or friends and had no personal stake in the collected data.

Conclusion

While action research has been used extensively in the health sector, there has been little evidence of its relevance to promoting male involvement in maternal and child health. This

For peer review only - http://bmjopen.bmj.com/site/about/guidelines.xhtml

study is one of the few to have used action research approach to promote male involvement in maternal and child health. We have demonstrated how stakeholders, including men, were able to design culturally acceptable strategies to increase male involvement in maternal and child health. The use of participatory approach empowered communities, including women, to diagnose barriers to male involvement and develop culturally acceptable strategies to address these barriers. Our participatory approach to male involvement increases the sustainability of the interventions beyond the life span of the project. The IMCHA programme is now going into the last phase of implementation and evaluation of effects of these strategies on maternal and child health outcomes. More lessons will be identified during the implementation of these strategies.

Acknowledgements
This study was part of the Innovating for Maternal and Child Health in Africa (IMCHA) programme, which is being implemented in Kilolo and Mufindi districts in Iringa Region, Tanzania. We are grateful to the regional, district and local level stakeholders for participating in this study.

Authors' contribution
SM, PK, KB & MA conceptualised the study, including developing data collection tools. SM, PK, JP collected, coded and analysed data. JP & SM drafted the manuscript. PK, SF, KB & MA contributed to the manuscript writing. All authors approved the final manuscript.

Funding Information
The study was funded by a joint initiative of three Canadian government agencies, namely; the Canadian Institutes of Health Research (CIHR), Global Affairs Canada and Canada's International Development Research Centre (IDRC) under IMCHA programme Grant No: IDRC 108023-001

Competing interests
The authors declare that they have no conflict of interest.

Ethics Approval
This study got approval from the Ethical Committee of the Medical Research Council of Tanzania (NIMR) No: NIMR/HQ/R.8a/Vol.IX/2119. The study was also approved by the Regional Administrative Officers in the region of study.

Availability of data
The datasets are not publicly available since participants did not give consent for the public sharing of their information. However, summaries of the information and data collection tools are available from the corresponding author upon request.

REFERENCES

1. World Health Organization, UNICEF, United Nations, Department of Economic and Social Affairs, Population Division, World Bank. Trends in maternal mortality: 1990 to 2015: estimates by WHO, UNICEF, UNFPA, World Bank Group and the United Nations Population Division [Internet]. 2015 [cited 2017 Feb 3]. Available from: http://www.who.int/reproductivehealth/publications/monitoring/maternal-mortality-2015/en/.

For peer review only

2. Alkema L, Chou D, Hogan D, Zhang S, Moller A-B, Gemmill A, et al. Global, regional, and national levels and trends in maternal mortality between 1990 and 2015, with scenario-based projections to 2030: a systematic analysis by the UN Maternal Mortality Estimation Inter-Agency Group. Lancet Lond Engl. 2016 Jan 30;387(10017):462–74.
3. Althabe F, Bergel E, Cafferata ML, Gibbons L, Ciapponi A, Alemán A, et al. Strategies for improving the quality of health care in maternal and child health in low-and middle-income countries: an overview of systematic reviews. Paediatr Perinat Epidemiol. 2008;22(s1):42–60.
4. Tanzania Demographic and Health Survey. Tanzania Demographic and Health Survey and Malaria Indicator Survey 2015-2016. Dar es Salaam, Tanzania, and Rockville, Maryland, USA: 2016.
5. Afnan-Holmes H, Magoma M, John T, Levira F, Msemo G, Armstrong CE, et al. Tanzania's Countdown to 2015: an analysis of two decades of progress and gaps for reproductive, maternal, newborn, and child health, to inform priorities for post-2015. Lancet Glob Health. 2015;3(7): e396–e409.
6. Comrie-Thomsons L, Luchter S, Davis J, Mavhu W, Makungu C, Khan R, et al. Men Matter: Engaging Men for Better MNCH Outcomes. Burn Inst. 2015;
7. Mrisho M, Obrist B, Schellenberg JA, Haws RA, Mushi AK, Mshinda H, et al. The use of antenatal and postnatal care: perspectives and experiences of women and health care workers in rural southern Tanzania. *BMC Pregnancy Childbirth*. 2009 Dec 1;9(1):10.
8. Lassi ZS, Salam RA, Das JK, Bhutta ZA. Essential interventions for maternal, newborn and child health: background and methodology. Reprod Health. 2014;11(1): S1.
9. Thaddeus S, Maine D. Too far to walk: maternal mortality in context. *Soc Sci Med* 1982. 1994 Apr;38(8):1091–110.
10. Singh D, Lample M, Earnest J. The involvement of men in maternal health care: cross-sectional, pilot case studies from Maligita and Kibibi, Uganda. *Reprod Health*. 2014;11(1):68.
11. Redshaw M, Henderson J. Fathers' engagement in pregnancy and childbirth: evidence from a national survey. BMC Pregnancy Childbirth [Internet]. 2013 Dec [cited 2018 Mar 2];13(1). Available from: http://bmcpregnancychildbirth.biomedcentral.com/articles/10.1186/1471-2393-13-70.
12. Kiernan K, Pickett KE. Marital status disparities in maternal smoking during pregnancy, breastfeeding and maternal depression. *Soc Sci Med*. 2006 Jul;63(2):335–46.
13. Martin LT, McNamara MJ, Milot AS, Halle T, Hair EC. The Effects of Father Involvement during Pregnancy on Receipt of Prenatal Care and Maternal Smoking. *Matern Child Health J*. 2007 Oct 30;11(6):595–602.
14. Mekonnen W, Worku A. Determinants of low family planning use and high unmet need in Butajira District, South Central Ethiopia. Reprod Health [Internet]. 2011 Dec [cited 2018 Mar 2];8(1). Available from: http://reproductive-health-journal.biomedcentral.com/articles/10.1186/1742-4755-8-37.
15. Yue K, O'Donnell C, Sparks PL. The effect of spousal communication on contraceptive use in Central Terai, Nepal. *Patient Educ Couns*. 2010 Dec;81(3):402–8.
16. Barker G. A Radical Agenda for Men's Caregiving. *IDS Bull*. 2014;45(1):85–90.
17. Comrie-Thomson L, Tokhi M, Ampt F, Portela A, Chersich M, Khanna R, et al. Challenging gender inequity through male involvement in maternal and newborn health: critical assessment of an emerging evidence base. *Cult Health Sex*. 2015 Oct 16;17(sup2):177–89.
18. Kululanga LI, Sundby J, Malata A, Chirwa E. Striving to promote male involvement in maternal health care in rural and urban settings in Malawi: A qualitative study. *Reproductive Health* 2011; 8 (1): 36.

For peer review only - http://bmjopen.bmj.com/site/about/guidelines.xhtml

19. Mkandawile E, Hendriks SL. A qualitative Analysis of men's involvement in maternal and child health as a policy intervention in rural central Malawi. *BMC Pregnancy and Childbirth,* 2018; 18:37.
20. Mamba KC, Muula AS, Stones W. Facility-imposed barriers to early utilization of focused antenatal care services in Mangochi District, Malawi - a mixed methods assessment. *BMC Pregnancy and Childbirth*, 2017; 17:444.
21. Mgata S, Maluka SO. Factors for Late Initiation of Antenatal Care in Dar es Salaam, Tanzania: A Qualitative Study, *BMC pregnancy and Childbirth*, 19:415.
22. Peneza AK, Maluka SO. "Unless you come with your partner you will be sent back home": Strategies used to Promote Male Involvement in Antenatal Care in Southern Tanzania. *Global Health Action.*2018;11:1,1449724.
23. Pafs J, Binder-Finnema P, Klingberg-Allvin M, Rulisa S, Essen B. 'They would never receive you without a husband': Paradoxical barriers to antenatal care scale-up in Rwanda. *Midwifery*. 2015; (31): 1149-1156.
24. Ministry of Health. Focused antenatal care, malaria and syphilis in pregnancy: orientation package for service providers. Dar es Salaam, Tanzania; 2002.
25. Ministry of Health and Social Welfare. Guidelines for HIV testing and counselling in clinical settings. Dar es Salaam, Tanzania; 2007.
26. Tanzania Ministry of Health and Social Welfare. The National Road Map Strategic Plan to Improve Reproductive, Maternal, Newborn, Child & Adolescent Health in Tanzania (2016 - 2020) [Internet]. Ministry of Health and Social Welfare; 2015 [cited 2017 Jul 10]. Available from: http://ihi.eprints.org/3733/.
27. Maluka SO, Peneza AK. Perceptions on male involvement in pregnancy and childbirth in Masasi District, Tanzania: a qualitative study. *Reproductive Health*, 2018;15:68.
28. Aborigo RA, Reidpath DD, Oduro AR, Allotey P. Male involvement in maternal health: perspectives of opinion leaders. *BMC Pregnancy and Childbirth*, 2018; 18:3.
29. Ditekemena J, Koole O, Engmann C *et al*. Determinants of male involvement in maternal and child health services in sub-Saharan Africa: a review. *Reproductive Health*, 2012; 9:32.
30. Tokhi M, Comrie-Thomson L, Davis J, Portela A, Chersich M, Luchters S. Involving men to improve maternal and newborn health: A systematic review of the effectiveness of interventions. van Wouwe JP, editor. *PLOS ONE*. 2018; 25;13(1): e0191620.
31. Cornwall A, Jewkes R. What is participatory research? *Social Science & Medicine,* 1995; 41(12), 667–1676.
32. Reason P, Bradbury H. The Sage handbook of action research: participative inquiry and practice. 2nd ed. London; Thousand Oaks, Calif.: SAGE Publications; 2008.
33. Braun V, Clarke V. Using thematic analysis in psychology. *Qualitative Research in Psychology*, 2006; 3(2): 77-101.
34. Maluka S, Kamuzora P, Ndawi B, and Hurtig AK. Involving decision makers in the research process: Challenges of implementing accountability for reasonableness approach to priority setting at district level in Tanzania, *Global Public Health Journal*. 2014; 9 (7): 760-772.
35. Kamuzora P, Maluka S, Ndawi B, Byskov J, and Hurtig AK. Promoting community participation in priority setting in district health systems: Experiences from Mbarali district Tanzania, *Global Health Action*. 2013; 6: 22669.
36. Martineau T, Raven J, Aikins M, Alonso-Garbayo A, Baine S, Huss R, Maluka S, Wyss K. Strengthening health district management competencies in Ghana, Tanzania and Uganda: lessons from using action research to improve health workforce performance. *BMJ Global Health*. 2018; 3.

For peer review only - http://bmjopen.bmj.com/site/about/guidelines.xhtml

37. Colbourn, T., Nambiar A. & Costello A. The impact of quality improvement at health facilities and community mobilisation by women's groups on birth outcomes: an effectiveness study in three districts in Malawi. *MaiKhanda Final Evaluation Report,* Blantyre: The Health Foundation; 2013.
38. Lewycka S, Mwansambo C, Kazembe P, et al. A cluster randomised controlled trial of the community effectiveness of two interventions in rural Malawi to improve health care and to reduce maternal, newborn and infant mortality. *Trials*. 2010; 11:88.
39. Tripathy et al. Community mobilisation with women's groups facilitated by Accredited Social Health Activists (ASHAs) to improve maternal and newborn health in underserved areas of Jharkhand and Orissa: study protocol for a cluster-randomised controlled trial. *Trials.* 2011; 12:182.
40. Ekirapa-Kiracho E, Namazzi G, Tetui M, Mutebi A, Waiswa P, Oo[4+]H, Peters[4+]DH, George AS. Unlocking community capabilities for improving maternal and newborn health: participatory action research to improve birth preparedness, health facility access, and newborn care in rural Uganda. *BMC Health Services Research*. 2016;16(Suppl7): 638.
41. Kunene B, Beksinska M, Zondi S, *et al*. Involving Men in Maternity Care: South Africa. Durban, South Africa, Reproductive Health Research Unit in Durban, Department of Obstetrics and Gynecology, University of the Witwatersrand; 2004.
42. Bwambale FM, Ssali SN, Byaruhanga S, *et al*. Voluntary HIV counselling and testing among men in rural western Uganda: implications for HIV prevention. *BMC Public Health*, 2008; 8:263.
43. Mullany BC, Lakhey B, Shrestha D, *et al*. Impact of husbands' participation in antenatal health education services on maternal health knowledge. *Journal of Nepal Medical Association* 2009; 48:28-34.
44. Burke M, Rajabu M, J. B. Maximising male participation in PMTCT programs in Tanzania. International AIDS Conference, 11–16 July 2004; 15; abstract no. ThPeE8144.2004.Available :http://www.iasociety.org/Default.aspx?pageId=11&abstractId=2175807[cited 10 April 2018].
45. Kadomoto N, Iwasa H, Takashi M, Dulnuan MM, Kai I. Ifugao males, learning and teaching for the improvement of maternal and child health status in the Philippines: an evaluation of a program. *BMC Public Health*. 2011;11(280):1-10.
46. Manda-Taylor L, Mwale D, Phiri T, *et al*. Changing times? Gender roles and relationships in maternal, newborn and child health in Malawi. *BMC Pregnancy and Childbirth*, 2017; 17:321.
47. Oguntunde O, Surajo IM, Dauda DS, Salihu A, Anas-Kolo S, Sinai I. Overcoming barriers to access and utilization of maternal, newborn and child health services in northern Nigeria: an evaluation of facility health committees. *BMC Health Services Research.* 2018; 18: 104.
48. Ifakara Health Institute (IHI). Health facility committees: are they working? *Spotlight*. 2011, Issue 7.
49. Kessy FL. Improving Health Services through Community Participation in Health Governance Structures in Tanzania. *Journal of Rural and Community Development*. 2014; 9: 14-31.
50. Maluka S, Bukagire G. Community Participation in the Decentralised District Health Systems in Tanzania: Why do some Health Committees Perform better than others? *International Journal of Health Planning and Management.* 2016; 31(2): E86-E104.

For peer review only - http://bmjopen.bmj.com/site/about/guidelines.xhtml

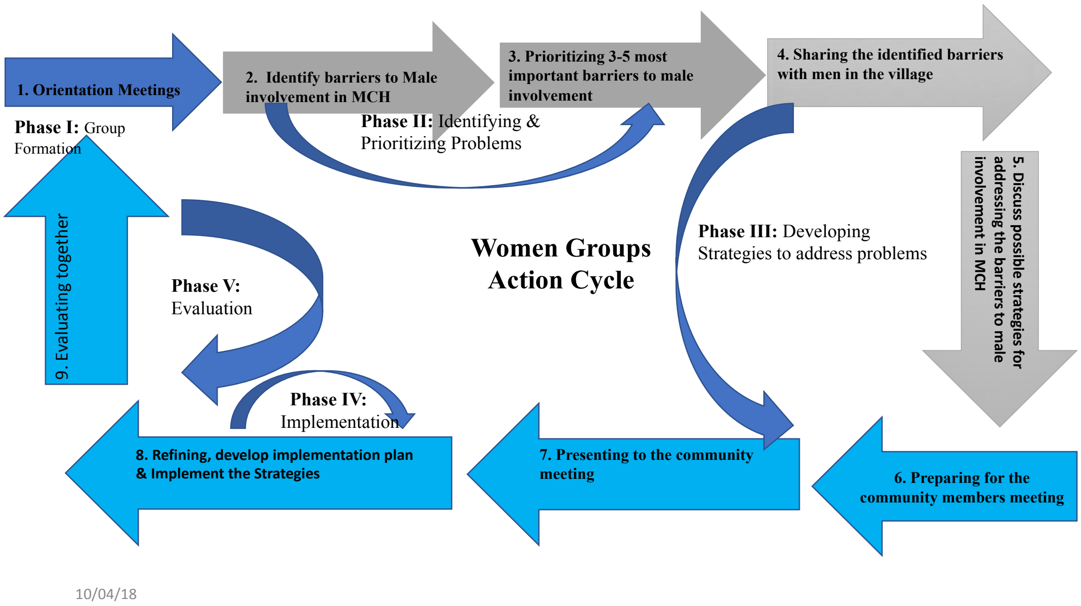

Figure 1: Participatory Action Research Cycle

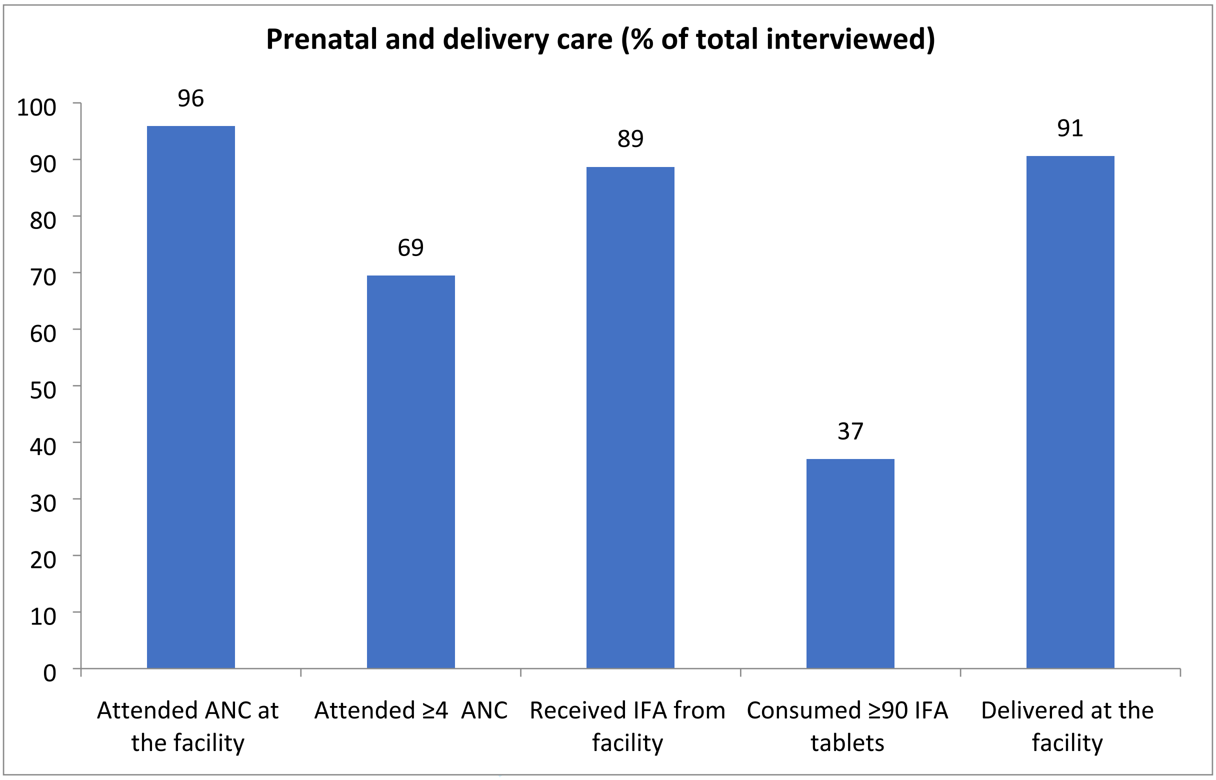

Figure 2: Prevalence of prenatal and delivery care among women group members

For peer review only

BMJ Open

Leaving no one behind: using action research to promote male involvement in maternal and child health in Iringa region, Tanzania

Journal:	*BMJ Open*
Manuscript ID	bmjopen-2020-038823.R1
Article Type:	Original research
Date Submitted by the Author:	18-Aug-2020
Complete List of Authors:	Maluka, Stephen; University of Dar es Salaam, Institute of Development Studies<br>Japhet, Paul; University of Dar es Salaam, Institute of Development Studies<br>Fitzgerald, Sian; Healthbridge Foundation<br>Begum, Khadija; Health bridge Foundation<br>Alexander, May; Iringa Region, Health Department<br>Kamuzora, Peter; University of Dar Es Salaam, Institute of Development Studies
**Primary Subject Heading**:	Health services research
Secondary Subject Heading:	Global health
Keywords:	Antenatal < GENETICS, PUBLIC HEALTH, Maternal medicine < OBSTETRICS

SCHOLARONE™
Manuscripts

I, the Submitting Author has the right to grant and does grant on behalf of all authors of the Work (as defined in the below author licence), an exclusive licence and/or a non-exclusive licence for contributions from authors who are: i) UK Crown employees; ii) where BMJ has agreed a CC-BY licence shall apply, and/or iii) in accordance with the terms applicable for US Federal Government officers or employees acting as part of their official duties; on a worldwide, perpetual, irrevocable, royalty-free basis to BMJ Publishing Group Ltd ("BMJ") its licensees and where the relevant Journal is co-owned by BMJ to the co-owners of the Journal, to publish the Work in this journal and any other BMJ products and to exploit all rights, as set out in our licence.

The Submitting Author accepts and understands that any supply made under these terms is made by BMJ to the Submitting Author unless you are acting as an employee on behalf of your employer or a postgraduate student of an affiliated institution which is paying any applicable article publishing charge ("APC") for Open Access articles. Where the Submitting Author wishes to make the Work available on an Open Access basis (and intends to pay the relevant APC), the terms of reuse of such Open Access shall be governed by a Creative Commons licence – details of these licences and which Creative Commons licence will apply to this Work are set out in our licence referred to above.

Other than as permitted in any relevant BMJ Author's Self Archiving Policies, I confirm this Work has not been accepted for publication elsewhere, is not being considered for publication elsewhere and does not duplicate material already published. I confirm all authors consent to publication of this Work and authorise the granting of this licence.

For peer review only

Leaving no one behind: using action research to promote male involvement in maternal and child health in Iringa region, Tanzania

Stephen Oswald Maluka[1*], Paul Japhet [2], Sian Fitzgerald[3], Khadija Begum[3], May Alexander[4] & Peter Kamuzora[2]

1. University of Dar es Salaam College of Education (DUCE), P.O.BOX 2329, Dar es Salaam, Tanzania
2. Institute of Development Studies, University of Dar es Salaam. P.O.BOX 35169, Dar es Salaam, Tanzania
3. Health bridge Foundation of Canada.
4. Iringa Municipal Council, Health Department. Iringa, Tanzania

SOM: stephenmaluka@yahoo.co.uk
JP: pijsamba@yahoo.com
SF: sfitzgerald@healthbridge.ca
KB: khadijabgm@gmail.com
MA: may20tz@yahoo.com
PK: petkamu@Udsm.ac.tz

* Corresponding Author
SOM: stephenmaluka@yahoo.co.uk

For peer review only

For peer review only - http://bmjopen.bmj.com/site/about/guidelines.xhtml

ABSTRACT

Introduction

Male involvement has been reported to improve maternal and child health (MCH) outcomes. However, most studies in Low and Middle Income Countries (LMIC) have reported low participation of men in MCH related programmes. While there is a growing interest in the involvement of men in maternal and child health, little is known on how male involvement can be effectively promoted in settings where entrenched unequal gender roles, norms and relations constrain women from effectively inviting men to participate in maternal and child health.

Methods and analysis

This paper reports participatory action research aimed to promote male participation in pregnancy and childbirth in Iringa Region, Tanzania. As part of the Innovating for Maternal and Child Health in Africa (IMCHA) project, participatory Action Research (PAR) was conducted in 20 villages in two rural districts in Tanzania. Men and women were engaged separately to identify barriers to male involvement in antenatal care (ANC) and during delivery; and then they were facilitated to design strategies to promote male participation in their communities. Along with the PAR intervention, researchers undertook a series of research activities. A thematic analysis was used to analyse the data. The common strategies designed were: engaging health facility committees; using male champions and male gatekeepers; and using female champions to sensitise and provide health education to women. These strategies were validated during stakeholders' meetings, which were convened in each community.

Discussion

The use of participatory approach not only empowers communities to diagnose barriers to male involvement and develop culturally acceptable strategies but also increases sustainability of the interventions beyond the life span of the project. More lessons will be identified during the implementation of these strategies.

Strengths and Limitations

- This study is one of the few to have used action research approach to promote male involvement in maternal and child health.
- The findings reported are based on the initial stages of the participatory action research.
- Another limitation of the study is lack of independence of the data collection because data collection was carried out by the researchers who were part of the ART.

Key Words: Male involvement; Maternal and Child Health; Participatory Action Research; Tanzania

For peer review only - http://bmjopen.bmj.com/site/about/guidelines.xhtml

1. Introduction

Maternal and Child death is a global problem and is highly overwhelming in low and middle income countries. An estimate of 303,000 pregnant women and 8.4 million under-five children died in 2015 [1]. Low and middle-income countries account for approximately 99% of maternal and child deaths [2-3]. In Tanzania, the maternal mortality rate is 556 per 100,000 live births while the infant and under-five mortality rates are 43/1,000 and 67/1,000 respectively [4]. The neonatal mortality rate is 25 per 1,000 live births [4].

Low utilisation of maternal and child health services is mentioned as one of the root causes of maternal and child deaths [1-3]. In Tanzania, for example, though over 90% of pregnant women attend antenatal care (ANC) at least once, only 51% complete four or more recommended visits. Additionally, only 24% of women attend their first ANC before the fourth month of pregnancy [4-5]. Health facility deliveries were 63% and 37% were home deliveries. 64% of deliveries were assisted by health professional and 36% were not [4]. Furthermore, only 13% of women who delivered at home attended postnatal care (PNC) within the recommended timeline of 48 hours after delivery [4].

In Tanzania, like in other African countries, the adverse effect of socio-economic factors on maternal, new-born and child health is compounded by gender inequalities that limit women's autonomy and decision-making power [4]. This prevents women and their families from practicing healthy behaviours as well as seeking and receiving life-saving care at and around the time of birth [4, 6-9]. The delay in seeking care and delay in reaching healthcare facilities occurs when women lack support from male partners, especially when the situation involves the need for money [9-10].

Male involvement has been reported to improve maternal and child health outcomes. Among the perceived benefits associated with increased male involvement include increased access to ANC and delivery at the health facility with assistance of skilled birth attendants [11]; increased knowledge of both women and men on the obstetric care emergencies [12-13]; increased use of family planning methods [14-15]; and decreased gender-related barriers to maternal and child health services [16-17].

Globally, a number of strategies have been implemented to increase male involvement in maternal and child health services. The strategies include health education campaigns on the mass media, at work places, community outreach, and counselling of couples at health facilities [18-19]. Others are fast tracking to couples who attend clinics together [18-22] and denying services to women attending ANC without male partners [19-23].

In Tanzania, policies, guidelines and strategies emphasize male involvement in maternal and child health services. The focused antenatal care (FANC) guidelines require pregnant women to attend clinics with their partners as part of the prevention of mother to child transmission (PMTCT) of Human Immunodeficiency Virus (HIV). Pregnant women are, therefore, required to attend the first ANC visits with their male partners for HIV testing [24-26]. Despite these strategies, only 30% of male partners participate in the couple counselling and HIV testing [26].

While there is a growing interest in the involvement of men in maternal and child health, little is known on how male involvement can be effectively promoted in settings where entrenched

For peer review only - http://bmjopen.bmj.com/site/about/guidelines.xhtml

unequal gender roles, norms and relations constrain women from effectively inviting men to participate in maternal and child health. Most policies take a narrow definition of male involvement focusing more on clinic attendance. In this study, male involvement refers to provision of social, informational and psychological support to pregnant women. This paper makes a valuable contribution to the literature on male involvement in maternal and child health because it addresses the question of how male involvement can be promoted.

2. Methods

2.1 Study Settings and Design

This study was part of the large project under the Innovating for Maternal and Child Health in Africa (IMCHA) programme, which is being implemented in Kilolo and Mufindi districts in Iringa region, Tanzania. IMCHA programme (September 2015- March 2020) aimed to improve maternal, new-born and child health outcomes by strengthening health systems to become more equitable, using primary health care as an entry point. Iringa region was selected because of the earlier research collaboration between some of the IMCHA researchers and the regional and district decision makers on strengthening decentralised district health management. The Districts are predominantly rural and the majority of the population rely on agriculture for subsistence and production. Table 1 shows maternal and child health indicators in the study Districts.

T*able 1: Maternal and child health indicators in the study Districts*

Indicator	National (DHS 2015/16)	Kilolo (HMIS)	Mufindi (HMIS)
ANC visits within 12 weeks	24%	27%	16.8%
Four or more ANC visits	51%	27.1%	23.6%
Facility delivery	63%	92.2%	96.8%
Assisted by skilled birth attendants	64%	92%	92%
Postnatal care within 48 hours	31%	76.2%	51.8%
Male involvement in ANC HIV testing	30%	Low	Low
Use of Morden Family Planning	32%	30%	30%

DHS - Demographic and Health Survey; **HMIS** - Health Management Information System

A participatory action research (PAR) design was adopted in this study [27]. Action research is the research conducted in partnership with members of the community mainly aimed at bringing about structural or cultural change [27-28]. In PAR, researchers do not just conduct studies on community rather they form partnerships with community members to identify issues important to the local community, develop ways of studying them and take actions on the resulting knowledge [28]. The PAR process consisted of a series of meetings during which women, men and community stakeholders identified barriers to male involvement in maternal and child health; and then designed strategies to promote male participation. The Action Research Team (ART) led by the researchers from the University of Dar es Salaam, in collaboration with the regional and district health managers from Iringa Region facilitated the PAR. As indicated in Figure 1, the PAR process involved five phases. As indicated in Annex

For peer review only - http://bmjopen.bmj.com/site/about/guidelines.xhtml

1, in order to complete one cycle Women and men groups conducted 8 meetings on a monthly basis.

Figure 1: Participatory Action Research Cycle

2.1.1 Introduction and Formation of Women and Men Groups

We conducted a series of consultation meetings with the community members in 20 intervention villages. Prior to our first community, we had several discussions within our team of how our social identities with respect to education, gender, sexual orientation and material resources would influence the PAR process. This was an important process as most of the research team members had not worked with communities. Before starting community meetings, we conduct pre-visits in all 20 intervention Villages to discuss our research with community leaders. On entering the community, the ART first introduced the project to the community and facilitated the formation of women and men groups in 20 intervention villages. We used community leaders mainly Village Chairperson and Village Executive Officers to persuade other community members to attend an initial meeting. In each Village, women and men groups comprised 20 and 10 members respectively, who were selected during community meetings facilitated by the ART. The criteria for selecting women included: age 15-49 years, experience in utilizing MCH services, and women who were pregnant or had given birth in the last 12 months preceding the study. However, in a few Villages women who were above 50 years were selected by community members. These were mostly traditional birth attendants and the community considered them important to be included in the women groups in order to share their experience. The criteria for selecting male participants included: having a wife/female partner and experience with childbirth. In total, 400 women and 200 men were identified in 20 intervention Villages. These 400 women and 200 men made up several groups of 20 and 10 respectively. In addition, 40 community health workers (CHWs); two (2) from each village were identified and included in the women groups. The selected women, men and CHWs were given training on how they could diagnose various barriers to the access and use of maternal and child health services in their community; and then design strategies to address these barriers. The training was standardised and was provided by the experienced qualitative researchers (SOM & PK) in collaboration with the District health managers.

2.1.2 Diagnosing and prioritizing problems

In Phase II, the ART led the women groups in identifying and prioritising barriers to male involvement in maternal and child health in their community. This phase involved 2 different meetings. In the first meeting, women were able to identify barriers without the presence of men. In the next meeting, the ART facilitated women groups to share and discuss the identified barriers with a group of 10 men (male champions) in their community. The rationale of including men in the meetings was to give them an opportunity to share their views as key stakeholders and decision makers in maternal and child health matters. This process also aimed to prepare men to actively participate in designing and implementation of the strategies.

2.1.3 Designing male engagement strategies

In Phase III, women and men together developed strategies to promote male involvement in maternal and child health. In addition, the ART facilitated community meetings where women groups and male champions shared the barriers and strategies designed to promote male involvement. The key stakeholders who participated in the community meetings included the government and community leaders, religious leaders, health providers and members of the Health Facility Governing Committees. The objective of the community meetings was to give

For peer review only - http://bmjopen.bmj.com/site/about/guidelines.xhtml

stakeholders an opportunity to discuss and refine the problems, root causes and strategies identified by women and men groups. In addition, the community meetings aimed at actively involving community stakeholders in the implementation of the strategies in their respective areas. As indicated in Table 2, a total of 450 stakeholders from all 20 intervention villages participated in community meetings.

Table 2: Participants by type attending community meetings

**Districts**	**Kilolo**	**Mufindi**	**Total**
District Representatives	3	2	**5**
Ward Councillors	5	4	**9**
Ward Executive Officers	5	5	**10**
Community Development Officers	3	1	**4**
Village Executive Officers	8	10	**18**
Village Chair persons	10	9	**19**
Religious Leaders	18	19	**37**
Hamlet Chairs (community leaders)	52	49	**101**
Primary School Representatives	6	11	**17**
Secondary School Representatives	4	1	**5**
Health Facility Staff (health care workers)	5	4	**9**
Health Facility Governing Committees	4	4	**8**
Community Health Workers	20	18	**38**
Male Champions (men's representatives)	31	19	**50**
Women Groups Representatives	53	51	**104**
Others	11	5	**16**
	**238**	**212**	**450**

2.1.4 Implementing and evaluating strategies

In Phase IV, women and men groups developed plans and led the implementation of the strategies to promote male involvement in maternal and child health. In Phase V, women groups and male champions together evaluated the effectiveness of the strategies. In this paper, we report the first three phases of the PAR as the last two phases (implementation and evaluation) were not yet completed. As indicated in Table 3, in total, we facilitated 102 meetings between February 2017 and March 2018 for the first three phases.

For peer review only - http://bmjopen.bmj.com/site/about/guidelines.xhtml

Table 3: Meetings facilitated by the ART in the communities

	Type of meeting	*Number*
1	Orientation and formation of women and men groups	20
2	Training of women group members to identify barriers and design strategies to address these barriers	10
3	Training of Community Health Workers to supervise women and men in identifying barriers, designing strategies, implementation and evaluation of the strategies	2
4	Identification and prioritisation of barriers to MCH, including male involvement	20
5	Sharing identified barriers with men and jointly designing strategies	20
6	Preparing women group members for the community meetings (meeting logistics, preparing presentations, socio-dramas, role plays etc)	20
7	Community/stakeholders meetings to discuss and refine barriers, root causes and strategies	10
	**Total meetings**	**102**

2.2 Data collection techniques

As part of the PAR intervention, the researchers conducted a series of research activities. All the meetings conducted with women, men and community members were documented. In some cases, the discussions were audio-recorded with permission from the participants. Where recording was not possible, notes from the discussion were taken. In addition, trained research assistants prepared reports for the meetings. Similarly, all the presentations made by the women groups during community meetings were audio-recorded. Additionally, interviews and structured questionnaires were administered to a sub-set of men and women group members. Furthermore, a structured questionnaire was used to collect data on socio-demographic characteristics, men's knowledge and awareness of family planning and ANC services and their practices in relation to prenatal care and childbirth, and related decision making power. Data were collected through digital tablets using Open Data Kit (ODK) software by five local interviewers between June and July 2018. The same interviewers covered all 20 Villages. Researchers got verbal consent from all the prospective respondents. Verbal consent was preferred because in these settings the use of written consent forms might be perceived by participants to be threatening.

2.3 Data analysis

All recordings from the women, men and community meetings were transcribed verbatim by trained transcribers. The transcripts were reviewed by the core research team members and notes were made for each transcript. The transcripts and summaries of the stakeholders' meetings were analysed using a thematic approach [29]. First, the Principal Investigator (SOM) developed a code manual based on the objectives of the study. The coding framework was further discussed and refined by the research team members. PJ and SOM manually coded the transcripts and summaries of the meetings to the identified codes. Additional codes, which emerged during coding process, were added along the way. PK cross-checked the coding process. Responses were then compared across different categories of participants. Finally, data were summarized and synthesized, keeping the key expressions of respondents as illustrative cases. Quantitative data analyses were performed using Stata version 15.0 (Stata Corporation, College Station, TX, USA).

For peer review only - http://bmjopen.bmj.com/site/about/guidelines.xhtml

2.4 Patient and Public Involvement

This study was conducted in partnership with members of the community. The participatory action research process consisted of a series of meetings during which women, men and community stakeholders identified barriers to male involvement in maternal and child health and then designed strategies to promote male participation. Findings were shared during stakeholders meetings held in the respective communities.

3. Findings

This section presents the key findings of the study. The key themes covered are: characteristics of women and men groups, barriers to male involvement in maternal and child health and strategies designed to promote male involvement.

3.1.1 Characteristics of the women group members

The mean age (±SD) of the women respondents was 35.7±9.4 (Median=35, Min=16 and Max=61). Most of the women (75%) were between 26 and 49 years of age. About 78% (n=208) of the women were married, and most of them were living with their husbands (96%). Among the married women, 21% (n=44) reported that their husbands had more than one wife. Table 4 shows the main characteristics of the women group members.

Table 4: Characteristics of the women respondents

Characteristics	N	%
**Age category**		
16 to 25 y	41	15.5
26 to 35 y	98	37.0
36 to 49 y	100	37.7
50 to 65 y	26	9.8
**Marital status**		
Never married	29	10.9
Married	208	78.5
Divorced/separated	3	1.1
Widows	25	9.4
**Highest level of education**		
Never attended school	6	2.3
Primary, not completed	15	5.7
Primary, completed	191	72.1
Secondary, not completed	22	8.3
Secondary, completed	28	10.6
Vocational/ adult education	3	1.1

In terms of antenatal care and childbirth, around 9% (23 of 265) of the women group members were pregnant during the interviews, and 19 (83%) of them reported to attend ANC. We also explored prenatal and childbirth care practices of these women during their immediate pregnancy. Although around 96% of the women (254 of 265) attended antenatal care at health facilities, only about 72% of them (184 of 254) had had four or more ANC visits, and 93% (235 of 254) gave birth at health facilities. The prevalence of ≥4 ANC visits and facility births were low among all the women interviewed - 69% and 91% respectively.

For peer review only - http://bmjopen.bmj.com/site/about/guidelines.xhtml

3.1.2 Characteristics of male champions

As indicated in Table 5, about 51% (90) of the male champions were between the age range of 20-39. Moreover, 82% (144) had completed primary education and nearly all (98%) were married. As for women group members, the majority (92%) of the male champions relied on agriculture for subsistence and production.

Table 5: Male demographic characteristics

Male demographic characteristics	n	%
Age Range		
20-39	90	51
40-59	79	45
Above 60	6	3
Level of Education		
Not completed Primary school	2	1
Completed Primary school	144	82
Not completed Secondary School	11	6
Completed secondary School	15	9
College/University	3	2
Marital status		
Single	2	1
Married	172	98
Widower	1	1
Occupation		
Employed	2	1
Farmer	161	92
Self-employed/small business	12	7
Number of children		
1-3	85	49
4-6	69	39
7+	21	12

For peer review only

We also assessed the awareness and knowledge of male champions on various maternal and child health matters. As indicated in Table 6, nearly all participants (99%) were aware of the family planning (FP). However, most men (65%) only mentioned condom as the main FP method. Other methods of FP planning were rarely mentioned. The majority of the participants (84%) understood the role of ANC as taking care of pregnant women and their foetus. However, only 21% (63 of 175) agreed that men were required to accompany their wives for ANC visits. Furthermore, 80% (125 of 156) had knowledge of exclusive breastfeeding (EBF) and 78% (136 of 175) had knowledge of the duration of the EBF.

For peer review only - http://bmjopen.bmj.com/site/about/guidelines.xhtml

Table 6: Men's awareness and knowledge of maternal and child health matters

Awareness and knowledge of male champions on MCH	n	%
Ever heard of family planning		
Yes	173	99
No	1	1
Meaning of family planning		
Control of family size	62	26
Child spacing	145	61
Preventing unwanted pregnancy	32	13
Men's role in family planning		
Consent	54	27
Support	145	73
Others (Specify)	0	0
I don't know	0	0
Awareness of contraceptive methods for men		
Vasectomy	10	5
Male condom	144	65
Injectables	32	14
Diaphram	4	2
Hormonal contraception, such as the pill	32	14
What ANC entails		
Taking care of pregnant women and their foetus	162	84
Giving drugs and injection to pregnant women	9	5
Detecting and managing complications	21	11
Men's role in ANC		
Financial support	24	8
Encouraging and reminding pregnant women	105	36
Providing emotional and moral support	103	35
Accompanying pregnant women to clinic visits	63	21
Knowledge of exclusive breastfeeding (EBF)		
Breast milk alone	125	80
Breast milk with little water	3	2
No knowledge	28	18
Knowledge of the duration of EBF		
Below 6 months	13	7
6 months	136	78
Above 6 months	10	6
No knowledge	16	9

For peer review only

3.2 Barriers to male involvement

While majority of women preferred to attend first ANC appointment with their partners, men did not wish to be actively involved in ANC and delivery. Participants perceived men as breadwinners and their main role in pregnancy and child birth was to support their partners financially. There were contradicting views regarding the need for men to assist their wives

with household chores; while women preferred to be assisted with household chores during pregnancy and early days after birth, majority of male participants felt that this was mainly women's responsibility. Furthermore, it was evident that men did not prefer their wives to use modern family planning methods due to fear of side effects. Men also had the perception that the use of family planning would increase cheating for married women. Table 7 provides a summary of the barriers to male involvement as identified in most of the intervention villages.

Table 7: Barriers to male involvement in maternal and child health

Problem	Root Causes	Strategies to address root causes
Low male involvement in maternal and child health	Lack of health education, especially for men. Most often health education is provided by health workers during ANC clinics. Men rarely attended ANC clinics	-Male champions providing education to men during social gatherings<br>- Community and religious leaders providing education during community meetings
	Traditional gender roles and norms. Maternal and child health is considered as women's affair. Men's role is mainly supporting women financially	-Male champions providing education to men<br>-Community and religious leaders providing education during community meetings
	Fear of HIV testing. As part of the PMTCT programme, couples are required to test for HIV during ANC appointment. Men always prefer their wives to test first and assume that the wives' results would be the same to the husband.	-Male champions providing education to men on importance of couple HIV counselling and testing<br>-Community and religious leaders providing education during community meetings on importance of couple HIV counselling and testing
	Low birth spacing makes men fear to accompany their wives. Men think that they would be reprimanded by health providers and fellow men	-Male champions providing education to men on the importance of family planning<br>-Sensitizing health care to provide friendly services to couples attending clinics
	Couple relationships may encourage or hinder male participation in maternal and child health matters. The better the relationship, the higher the male participation and vice versa.	- Women group members sensitizing fellow women on the importance of better couple relationship
	Unfavourable environment in the health facilities. Most mentioned environments including long waiting time, disrespectful languages of the health providers, and lack of physical space to accommodate men and women concurrently.	-Health care workers to provide friendly services to couples attending clinics<br>-The local government to improve physical space to accommodate men and women concurrently<br>-Engaging health facility governing committees to improve health facilities

For peer review only - http://bmjopen.bmj.com/site/about/guidelines.xhtml

3.3 Strategies designed to promote male involvement

The key strategies which were commonly proposed were: engaging health facility governing committees; use of male champions and gatekeepers; and using women champions.

3.3.1 Engaging health facility governing committees

In all public primary health care facilities, there are health facility governing committees. The committees are composed of representatives of the community and health facility. The health facility governing committees have the mandate of overseeing the delivery of quality health care services and mobilizing the community to finance and manage health care services. The use of health facility governing committees was identified as a potential strategy to promote male engagement in maternal and child health services. Participants reported that actively engaging health facility governing committees would help address health facility related barriers to male involvement including long waiting time, disrespectful languages of the health providers, and lack of physical space to accommodate men and women concurrently as exemplified by one of the stakeholders:

> *The health governing committee should be coached on their roles and responsibilities to ensure they properly supervise provision of services at the health facility. If they are engaged, the committee will be able to talk to the health care workers not to harass pregnant women and their partners. The committee can also tackle other supply side related problems which hinder use of health care services. This will attract men to attend clinics* (Male participant during stakeholder meeting).

3.3.2 Engaging male champions and gate keepers

There was consensus among participants that men do not accompany their wives to ANC clinics; and fear to use family planning methods because they lack education. While women most often get health education in health facilities, particularly during ANC visits, men rarely receive health education. In addition, traditional gender roles and norms hinder men's participation in maternal and child health matters. Men's perception of superiority makes it difficult for wives to pass the message from health providers to their husbands. Most often wives fear to discuss family planning, pregnancy, and child birth related matters with their husbands due to culturally entrenched power differences between wives and husbands. In this context, male champions are better positioned to educate their fellow men and ultimately break the entrenched cultural gender roles and norms. Participants suggested that male champions should be identified in each village. They should also be facilitated in terms of key messages to be delivered to men. Male champions should deliver key messages through bars, football matches and other social gatherings in the villages as explained by some participants:

> *Men have inherited the beliefs that they are superior to women. Women are voiceless in making and influencing decisions at the household. Even the young generation has inherited the same cultural beliefs. It is difficult for women to educate or communicate to men the information they receive from the health facilities. We need to identify male champions who will talk to their fellow men in their social gatherings* (Religious leader).

Other participants added:

> *Most of the maternal and child health programmes are directed to women. Men do not get opportunities to receive health information becuaes they rarely attend clinics. Unfortunately, when women get the knowledge, they cannot deliver to their partners. They always fear to talk to their husbands* (Female participant).

For peer review only - http://bmjopen.bmj.com/site/about/guidelines.xhtml

We request IMCHA project team to focus mostly on men so that we can get first-hand information instead of waiting for female partners. We have always been excluded when it comes to maternal and child health education. Women are given education in health facilities. Men rarely get these opportunities (Male participant).

Closely connected to the above, participants in various meetings underlined the importance of engaging male gate keepers particularly religious and community leaders. It was highlighted that religious and community leaders are highly respected in the community. They have the power to convey health information to men and women alike. According to our participants, male gatekeepers should be actively engaged in the sensitization campaigns or in providing education to men and women in their communities as explained by some respondents:

*We should think very carefully about people who will deliver messages or education to the community. Others are an obstacle and using them as champions will end up losing instead of gaining. People will not listen; instead they will start whispering. But if we use influential people like religious leaders, elderly, or government officials, we might be able to increase the acceptability of the messages (*Community leader).

Another respondent added:

Women have presented the truth. The challenge is to get the right people who will deliver the key messages to the community and men in particular. I think if we work with religious and community leaders, the messages can easily be accepted by men (Male participant).

Almost all participants requested the IMCHA project team to provide training to male champions and gatekeepers. They also requested to provide them with key messages that should be delivered to the community, particularly to men. The key messages should focus on the importance of men to attend ANC clinics along with their partners, birth preparedness including shared decision making during pregnancy and child birth and the importance of family planning use.

This sensitization and training should not stop today. Women have presented very important issues which need to be addressed in our communities. We request researchers to provide more training to men, religious, and community leaders so that they can deliver these messages to the community. We have various platforms in our communities which can be used to communicate this information (Government leader).

3.3.3 Women champions and education of fellow women

There was consensus among participants that relationships among couples may encourage or hinder male participation in maternal and child health matters; on the one hand, unfriendly relationship will hinder active participation of men. On the other hand, good couple relationship will facilitate male involvement in maternal and child health. In this context, the use of women groups was considered important, particularly to provide education to their fellow women on the best practices. It was reported that women have many social platforms where they could share and discuss the information related to couple relationships during pregnancy and child birth. Improved couple relationships will increase male participation in maternal and child health as elaborated by some participants:

Men are like children. When you need to talk to them, you should read their mind and feelings. If they are in bad mood don't say any thing to them, wait until they

For peer review only - http://bmjopen.bmj.com/site/about/guidelines.xhtml

> *feel better. We need to understand the moods of our husbands and the best occasion that men can listen and understand our needs* (Female participant).

Other participants added that:

> *We, women, have problems. When a man agrees to do some household chores, we still want to make it public. We speak to our friends, neighbours or in saloons that, you just make my hair slowly, no need for hurry; my husband is taking care of children…. Even food, I will find it already cooked. If men know this, they will never support you again* (Female participant).

> *The way a wife communicates information to the husband may result in participation or non participation in antenatal care clinics. Some women think that when they are pregnant it is the time to harass their husbands. Men need soft language, if you use harsh or rude language, we interpret it as a command. Usually, men need respect and love. If you show us respect and love, we are ready to accompany you to ANC clinics and assist in domestic chores* (Male participant).

Another participant had this to say:

> *Women are the root cause of the low male involvement because they do not communicate with their husbands in a polite language. For example, when I come back home and tell my wife, I need the night meal (to have sex) and the wife replies rudely that 'I'm not ready', I will keep quiet. If I get the same answer the following day….I may start thinking of alternatives, looking for other women to satisfy my sexual desire. So, women need to use soft language, and we can understand them and plan together* (Male participant).

2. Discussion

This study adds new knowledge on the strategies for promoting male involvement in maternal and child health in low-income and middle-income countries. It is one of the few to have used action research approach to promote male involvement in maternal and child health. While action research has been used extensively in the health sector in Tanzania [30-32] and in other low-income and middle-income countries [33- 36], there has been little evidence of its appropriateness in promoting male involvement in maternal and child health. We have demonstrated how stakeholders, including men, were able to design culturally acceptable strategies to increase male involvement in maternal and child health. The next section discusses the strategies suggested by the participants in the current study in the light of other studies in low-income and middle-income countries.

4.1 Health education and community sensitization

Health education and community sensitisation campaigns were widely suggested in many villages as an important strategy which can be used to promote male participation in maternal and child health. Participants felt that majority of community members, particularly men, lack maternal and child health education. While male champions who participated in the study had adequate knowledge of ANC care, family planning and exclusive breast feeding, this was only a small segment of the population. In addition, male champions were selected based on their interests in maternal and child health matters. It may be possible that most of the male champions had experience in maternal and child health matters. Health education interventions tailored to women and men have been effective in increasing knowledge, health seeking behaviour and raising awareness on issues related to maternal and child health [18, 37-39]. In our study, participants said that in order to make it sustainable, health education should be provided by health care workers and community health workers who already exist in the communities. They also preferred the campaigns to be integrated in the community and

For peer review only - http://bmjopen.bmj.com/site/about/guidelines.xhtml

For peer review only

political gatherings in their respective villages. Participants preferred that key messages be developed to facilitate the campaigns and sensitisation programmes.
Connected to the above, participants suggested the use of male champions and male gatekeepers to promote behaviour change among men. It was evident from the findings that while women had access to health education during ANC clinics, men rarely attend health facilities. However, due to entrenched gender power relations, most often women are not able to convey the health information received from health facilities to their husbands. A study in Tanzania showed that the traditional way of using women to convey health information to male partners was a barrier to male involvement because men preferred to receive the message through other men [40]. The use of male champions is an effective strategy to include and engage men by targeting men focused key messages on the importance of participating in maternal and child health. A study in the Philippines reported that using male peer educators improved participation of men in maternal and child health matters [41]. Similarly, recent studies in Malawi underscored the importance of male champions in facilitating men's participation in maternal and child health and challenging traditional gender norms [19, 42]. Men were targeted with messages focused on the benefits of utilising maternal and child health services [19,42]. However, while in the Malawian studies male champions delivered the key messages to men secretly [19], in our study, the participants suggested that male champions should discuss maternal and child health openly in the men's platforms including football matches, bars and other places where men meet. Furthermore, sensitization and support should also be provided to religious, traditional, and government leaders to spread these messages in various religious, political, and social gatherings. Studies in other Africa countries have highlighted the importance of engaging religious and traditional leaders in promoting male involvement in maternal and child health [18 -19,42].

Furthermore, participants underlined the importance of managing couple relationships in order to increase men's participation in MCH services. Female and male participants suggested that women should learn how to strategically manage their couple relationships in order not to deter male men from engaging in MCH. It is absolutely the case that men's ability and willingness to be involved in MCH is influenced by couple relationship dynamics [19,42]. However, in our study, it seems that participants recommended that the burden of emotional labour involved in managing couple relationships should fall exclusively to women, and not to men. For example, it is the women and not men who are responsible for communicating with "soft language". While this finding is an important contribution to the literature, it implies the dominance of unequal expectations of men and women in the society. Managing couple relationships should be the responsibility of both women and men.

4.2 Use of health facility governing committees

In our study, participants suggested the use of health facility governing committees to address facility related barriers to male involvement in maternal and child health. Health facility governing committees have been reported to significantly facilitate access to maternal and child health services [43]. However, in Tanzania, studies have indicated that while health facility committees exist in all public primary health care facilities, limited financial resources, limited capacity and skills, attitudes of health workers and lack of clarity of the role and mandate of committees affect the performance of the committees [44-46]. In order to maximise their performance, deliberate efforts should be made to strengthen the capacity of the health committees. Furthermore, the Ministry of Health should ensure that district Councils allocate adequate funds to the district annually to support meetings of the health committees as it is done for the council Health Services Boards (CHSBs). Studies have reported that CHSBs in Tanzania managed to attract members with good qualification mainly

For peer review only - http://bmjopen.bmj.com/site/about/guidelines.xhtml

due to the availability of good incentives [45-46].

Limitations of the study

The main limitation of this study is that it does not report the implementation and effects of the designed strategies. The findings reported are based on the initial stages of the participatory action research. The programme is now going into the fourth and fifth phases of implementation and evaluation of the effects of the strategies on male involvement in maternal and child health. Another limitation of the study is lack of independence of the data collection. The data collection was carried out by the researchers who were part of the ART. However, the data collectors had no motivation to consciously or subconsciously skew the responses in a particular direction, although errors in data collection will always be expected. The data collectors were paid to collect the data as accurately as possible and their compensation did not depend on what data they collected. Moreover, the interviewers did not collect data from their family or friends and had no personal stake in the collected data.

Conclusion

While action research has been used extensively in the health sector, there has been little evidence of its relevance to promoting male involvement in maternal and child health. This study is one of the few to have used action research approach to promote male involvement in maternal and child health. We have demonstrated how stakeholders, including men, were able to design culturally acceptable strategies to increase male involvement in maternal and child health. The use of participatory approach empowered communities, including women, to diagnose barriers to male involvement and develop culturally acceptable strategies to address these barriers. Our participatory approach to male involvement may likely increase sustainability of the interventions beyond the life span of the project. The IMCHA programme is now going into the last phase of implementation and evaluation of effects of these strategies on maternal and child health outcomes. More lessons will be identified during the implementation of these strategies.

Acknowledgements

This study was part of the Innovating for Maternal and Child Health in Africa (IMCHA) programme, which is being implemented in Kilolo and Mufindi districts in Iringa Region, Tanzania. We are grateful to the regional, district and local level stakeholders for participating in this study.

Authors' contribution

SM, PK, KB & MA conceptualised the study, including developing data collection tools. SM, PK, JP collected, coded and analysed data. JP & SM drafted the manuscript. PK, SF, KB & MA contributed to the manuscript writing. All authors approved the final manuscript.

Funding Information

The study was funded by a joint initiative of three Canadian government agencies, namely; the Canadian Institutes of Health Research (CIHR), Global Affairs Canada and Canada's International Development Research Centre (IDRC) under IMCHA programme Grant No: IDRC 108023-001

Competing interests

The authors declare that they have no conflict of interest.

For peer review only

Ethics Approval
This study got approval from the Ethical Committee of the Medical Research Council of Tanzania (NIMR) No: NIMR/HQ/R.8a/Vol.IX/2119. The study was also approved by the Regional Administrative Officers in the region of study.

Availability of data
The datasets are not publicly available since participants did not give consent for the public sharing of their information. However, summaries of the information and data collection tools are available from the corresponding author upon request.

For peer review only

REFERENCES

1. World Health Organization, UNICEF, United Nations, Department of Economic and Social Affairs, Population Division, World Bank. Trends in maternal mortality: 1990 to 2015: estimates by WHO, UNICEF, UNFPA, World Bank Group and the United Nations Population Division [Internet]. 2015 [cited 2017 Feb 3]. Available from: http://www.who.int/reproductivehealth/publications/monitoring/maternal-mortality-2015/en/.
2. Alkema L, Chou D, Hogan D, Zhang S, Moller A-B, Gemmill A, et al. Global, regional, and national levels and trends in maternal mortality between 1990 and 2015, with scenario-based projections to 2030: a systematic analysis by the UN Maternal Mortality Estimation Inter-Agency Group. Lancet Lond Engl. 2016 Jan 30;387(10017):462–74.
3. Althabe F, Bergel E, Cafferata ML, Gibbons L, Ciapponi A, Alemán A, et al. Strategies for improving the quality of health care in maternal and child health in low-and middle-income countries: an overview of systematic reviews. Paediatr Perinat Epidemiol. 2008;22(s1):42–60.
4. Tanzania Demographic and Health Survey. Tanzania Demographic and Health Survey and Malaria Indicator Survey 2015-2016. Dar es Salaam, Tanzania, and Rockville, Maryland, USA: 2016.
5. Afnan-Holmes H, Magoma M, John T, Levira F, Msemo G, Armstrong CE, et al. Tanzania's Countdown to 2015: an analysis of two decades of progress and gaps for reproductive, maternal, newborn, and child health, to inform priorities for post-2015. Lancet Glob Health. 2015;3(7): e396–e409.
6. Comrie-Thomsons L, Luchter S, Davis J, Mavhu W, Makungu C, Khan R, et al. Men Matter: Engaging Men for Better MNCH Outcomes. Burn Inst. 2015;
7. Mrisho M, Obrist B, Schellenberg JA, Haws RA, Mushi AK, Mshinda H, et al. The use of antenatal and postnatal care: perspectives and experiences of women and health care workers in rural southern Tanzania. *BMC Pregnancy Childbirth*. 2009 Dec 1;9(1):10.
8. Lassi ZS, Salam RA, Das JK, Bhutta ZA. Essential interventions for maternal, newborn and child health: background and methodology. Reprod Health. 2014;11(1): S1.
9. Thaddeus S, Maine D. Too far to walk: maternal mortality in context. *Soc Sci Med* 1982. 1994 Apr;38(8):1091–110.
10. Singh D, Lample M, Earnest J. The involvement of men in maternal health care: cross-sectional, pilot case studies from Maligita and Kibibi, Uganda. *Reprod Health*. 2014;11(1):68.
11. Redshaw M, Henderson J. Fathers' engagement in pregnancy and childbirth: evidence from a national survey. BMC Pregnancy Childbirth [Internet]. 2013 Dec [cited 2018 Mar

2];13(1). Available from: http://bmcpregnancychildbirth.biomedcentral.com/articles/10.1186/1471-2393-13-70.

12. Kiernan K, Pickett KE. Marital status disparities in maternal smoking during pregnancy, breastfeeding and maternal depression. *Soc Sci Med*. 2006 Jul;63(2):335–46.
13. Martin LT, McNamara MJ, Milot AS, Halle T, Hair EC. The Effects of Father Involvement during Pregnancy on Receipt of Prenatal Care and Maternal Smoking. *Matern Child Health J*. 2007 Oct 30;11(6):595–602.
14. Mekonnen W, Worku A. Determinants of low family planning use and high unmet need in Butajira District, South Central Ethiopia. Reprod Health [Internet]. 2011 Dec [cited 2018 Mar 2];8(1). Available from: http://reproductive-health-journal.biomedcentral.com/articles/10.1186/1742-4755-8-37.
15. Yue K, O'Donnell C, Sparks PL. The effect of spousal communication on contraceptive use in Central Terai, Nepal. *Patient Educ Couns*. 2010 Dec;81(3):402–8.
16. Barker G. A Radical Agenda for Men's Caregiving. *IDS Bull*. 2014;45(1):85–90.
17. Comrie-Thomson L, Tokhi M, Ampt F, Portela A, Chersich M, Khanna R, et al. Challenging gender inequity through male involvement in maternal and newborn health: critical assessment of an emerging evidence base. *Cult Health Sex*. 2015 Oct 16;17(sup2):177–89.
18. Kululanga LI, Sundby J, Malata A, Chirwa E. Striving to promote male involvement in maternal health care in rural and urban settings in Malawi: A qualitative study. *Reproductive Health* 2011; 8 (1): 36.
19. Mkandawile E, Hendriks SL. A qualitative Analysis of men's involvement in maternal and child health as a policy intervention in rural central Malawi. *BMC Pregnancy and Childbirth,* 2018; 18:37.
20. Mamba KC, Muula AS, Stones W. Facility-imposed barriers to early utilization of focused antenatal care services in Mangochi District, Malawi - a mixed methods assessment. *BMC Pregnancy and Childbirth*, 2017; 17:444.
21. Mgata S, Maluka SO. Factors for Late Initiation of Antenatal Care in Dar es Salaam, Tanzania: A Qualitative Study, *BMC pregnancy and Childbirth*, 19:415.
22. Peneza AK, Maluka SO. "Unless you come with your partner you will be sent back home": Strategies used to Promote Male Involvement in Antenatal Care in Southern Tanzania. *Global Health Action.*2018;11:1,1449724.
23. Pafs J, Binder-Finnema P, Klingberg-Allvin M, Rulisa S, Essen B. 'They would never receive you without a husband': Paradoxical barriers to antenatal care scale-up in Rwanda. *Midwifery*. 2015; (31): 1149-1156.
24. Ministry of Health. Focused antenatal care, malaria and syphilis in pregnancy: orientation package for service providers. Dar es Salaam, Tanzania; 2002.
25. Ministry of Health and Social Welfare. Guidelines for HIV testing and counselling in clinical settings. Dar es Salaam, Tanzania; 2007.
26. Tanzania Ministry of Health and Social Welfare. The National Road Map Strategic Plan to Improve Reproductive, Maternal, Newborn, Child & Adolescent Health in Tanzania (2016 - 2020) [Internet]. Ministry of Health and Social Welfare; 2015 [cited 2017 Jul 10]. Available from: http://ihi.eprints.org/3733/.
27. Cornwall A, Jewkes R. What is participatory research? *Social Science & Medicine,* 1995; 41(12), 667–1676.
28. Smith L, Bratini L, Chambers DA, Jensen RV, Romero LL. Between idealism and reality: Meeting the challenges of participatory action research. *Action Research*, 2010; 8 (4): 407-425.
29. Braun V, Clarke V. Using thematic analysis in psychology. *Qualitative Research in Psychology*, 2006; 3(2): 77-101.

For peer review only - http://bmjopen.bmj.com/site/about/guidelines.xhtml

30. Maluka S, Kamuzora P, Ndawi B, and Hurtig AK. Involving decision makers in the research process: Challenges of implementing accountability for reasonableness approach to priority setting at district level in Tanzania, *Global Public Health Journal*. 2014; 9 (7): 760-772.
31. Kamuzora P, Maluka S, Ndawi B, Byskov J, and Hurtig AK. Promoting community participation in priority setting in district health systems: Experiences from Mbarali district Tanzania, *Global Health Action*. 2013; 6: 22669.
32. Martineau T, Raven J, Aikins M, Alonso-Garbayo A, Baine S, Huss R, Maluka S, Wyss K. Strengthening health district management competencies in Ghana, Tanzania and Uganda: lessons from using action research to improve health workforce performance. *BMJ Global Health*. 2018; 3.
33. Colbourn, T., Nambiar A. & Costello A. The impact of quality improvement at health facilities and community mobilisation by women's groups on birth outcomes: an effectiveness study in three districts in Malawi. *MaiKhanda Final Evaluation Report,* Blantyre: The Health Foundation; 2013.
34. Lewycka S, Mwansambo C, Kazembe P, et al. A cluster randomised controlled trial of the community effectiveness of two interventions in rural Malawi to improve health care and to reduce maternal, newborn and infant mortality. *Trials*. 2010; 11:88.
35. Tripathy et al. Community mobilisation with women's groups facilitated by Accredited Social Health Activists (ASHAs) to improve maternal and newborn health in underserved areas of Jharkhand and Orissa: study protocol for a cluster-randomised controlled trial. *Trials.* 2011; 12:182.
36. Ekirapa-Kiracho E, Namazzi G, Tetui M, Mutebi A, Waiswa P, Oo[4+]H, Peters[4+]DH, George AS. Unlocking community capabilities for improving maternal and newborn health: participatory action research to improve birth preparedness, health facility access, and newborn care in rural Uganda. *BMC Health Services Research*. 2016;16(Suppl7): 638.
37. Kunene B, Beksinska M, Zondi S, *et al*. Involving Men in Maternity Care: South Africa. Durban, South Africa, Reproductive Health Research Unit in Durban, Department of Obstetrics and Gynecology, University of the Witwatersrand; 2004.
38. Bwambale FM, Ssali SN, Byaruhanga S, *et al*. Voluntary HIV counselling and testing among men in rural western Uganda: implications for HIV prevention. *BMC Public Health*, 2008; 8:263.
39. Mullany BC, Lakhey B, Shrestha D, *et al*. Impact of husbands' participation in antenatal health education services on maternal health knowledge. *Journal of Nepal Medical Association* 2009; 48:28-34.
40. Burke M, Rajabu M, J. B. Maximising male participation in PMTCT programs in Tanzania. International AIDS Conference, 11–16 July 2004; 15; abstract no. ThPeE8144.2004.Available :http://www.iasociety.org/Default.aspx?pageId=11&abstractId=2175807[cited 10 April 2018].
41. Kadomoto N, Iwasa H, Takashi M, Dulnuan MM, Kai I. Ifugao males, learning and teaching for the improvement of maternal and child health status in the Philippines: an evaluation of a program. *BMC Public Health*. 2011;11(280):1-10.
42. Manda-Taylor L, Mwale D, Phiri T, *et al*. Changing times? Gender roles and relationships in maternal, newborn and child health in Malawi. *BMC Pregnancy and Childbirth*, 2017; 17:321.
43. Oguntunde O, Surajo IM, Dauda DS, Salihu A, Anas-Kolo S, Sinai I. Overcoming barriers to access and utilization of maternal, newborn and child health services in

For peer review only - http://bmjopen.bmj.com/site/about/guidelines.xhtml

northern Nigeria: an evaluation of facility health committees. *BMC Health Services Research.* 2018; 18: 104.

44. Ifakara Health Institute (IHI). Health facility committees: are they working? *Spotlight*. 2011, Issue 7.
45. Kessy FL. Improving Health Services through Community Participation in Health Governance Structures in Tanzania. *Journal of Rural and Community Development*. 2014; 9: 14-31.
46. Maluka S, Bukagire G. Community Participation in the Decentralised District Health Systems in Tanzania: Why do some Health Committees Perform better than others? *International Journal of Health Planning and Management.* 2016; 31(2): E86-E104.

For peer review only

For peer review only - http://bmjopen.bmj.com/site/about/guidelines.xhtml

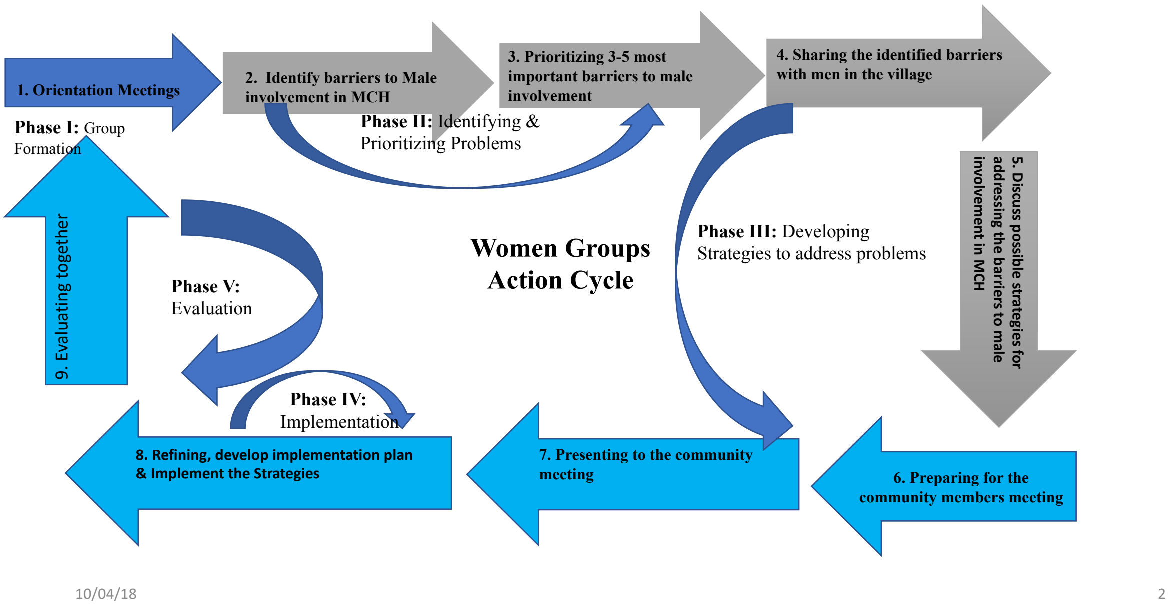

Figure 1: Participatory Action Research Cycle

Improving Access to Health Services and Quality of Care for Mothers and Children in Tanzania (IMCHA)

Women Group Meetings

By

Institute of Development Studies, University of Dar es Salaam,
Health Bridge Foundation of Canada
And
Regional Commissioner's Office (Health Department) of Iringa Region

May, 2017

For peer review only

The Women Groups will complete a cycle of problem identification and prioritization, developing strategies, implementation of the strategies and evaluating the strategies to improve maternal and child health in their village. In order to complete one cycle Women Groups will conduct 8 meetings on a monthly basis.

Meeting 1: Identifying maternal and neonatal problems in the community

- Through questions and answers collect the women group's maternal and neonatal problems:
- Place all the picture cards on the floor.
- Ask each women to select one.
- Ask each women in turn, to describe the problem which she sees in the picture, what it is called (if it has a particular name), what causes this problem
- Write down the descriptions in a table on newsprint (see example in the box).
- If there are any problems other than what is shown in the picture cards, please add.

Meeting 2: Sharing the identified problem with men in the village: The objective is to make men aware of the problems and provide opportunity for men to participate in the process.

Meeting 3: Prioritizing problems - To decide on the most important problems that need to be addressed

Activities

To decide 3-5 most important maternal and neonatal health problems that need to be addressed in the community.

Discuss problems which were identified in the previous meeting

- Ask which 3-5 are the most important maternal and neonatal problems, to try to deal with in the community?
- Discuss and arrive at a consensus of the 3-5 priority problems.

Discussion points:

- What are the most frequent problems that we see on the newsprint?
- What problems are directly related to maternal and neonatal health?
- Can we deal with these problems in the community?

Meeting 4: Discuss possible strategies for addressing the priority problems

Activities

- To discuss possible strategies for addressing the priority problems.
- Remind the women of the three priority problems, which were identified in the last meeting.
- With the first problem, ask, what could we do when this problem arises?
- Discuss the different strategies for this problem. Probing maybe needed.
- List the different strategies.
- Explore, what resources the community has to deal with this problem?
-

Possible Resources

Who	What can they do
Women's group	▪ Create awareness<br>▪ Create pressure<br>▪ Provide local equipment/labour

For peer review only

	▪ Plan, implement, monitor and evaluate the activities
IMCHA project	▪ Facilitate to create awareness<br>▪ Facilitate to mobilise group<br>▪ Co-ordinate and liaise with other organisations<br>▪ Strengthen the health institutions & committees
Community	▪ Participate in the different strategies<br>▪ Provide suggestions and feedback<br>▪ Provide public property, such as building, furniture, equipment, utensils<br>▪ Demand and create pressure
Health Facility	▪ Provide health service and treatment<br>▪ Provide medicine & medical supplies<br>▪ Run outreach and immunisation clinic<br>▪ Create awareness
Other organizations	▪ Provide medicines and equipment<br>▪ Organise health camp and clinics<br>▪ Support economically<br>▪ Provide training

- Discuss, and list the strategies and the resources and make realistic strategies.
- Repeat these steps for the second and third priority problems.

Meeting 5: Preparing for the community members meeting

Activities

To prepare for the community members meeting.

- **Discussion points**:
  - ✓ When will the community meeting take place?
  - ✓ Where will it take place?
  - ✓ Who will invite the participants?
  - ✓ How will they be invited? (verbally, by letter)
  - ✓ Who will be responsible for this?
- Discuss about preparing the verbal presentations. **Discussion points**:
  - ✓ Who can explain what the group did, at the community meeting?
  - ✓ Who can explain about the three problems, at the community meeting?
  - ✓ Who can explain about the strategies, at the community meeting?
  - ✓ What is the best way of presenting this information? (charts, drawings)
  - ✓ Should one woman explain all or should different women explain different things?
- Discuss about preparing the socio-drama
  - ✓ What should the drama be about?
  - ✓ Who will write the story?
  - ✓ Who will perform as actors?

Meeting 6: Presenting to the community meeting

Activities

- ***The community members will learn about what the women have been doing*** - The WGF needs to open the meeting, briefly introduce IMCHA project, and introduce the women, their work and the purpose of this meeting to the community members.
- ***The community members will learn about the three problems identified by the women-*** The women will present how the information on the problems was collected. The women need to present their three priority problems (verbal presentations OR

For peer review only

socio-drama OR both).

- ***The community members will learn about the possible strategies that the women have suggested to address the three problems*** - The women need to present their three problems and the possible strategies to address these problems.
- to reach a consensus of the strategies

Meeting 7: Refining and Implementation of the Strategies

- **Agree on the strategies-** The WGFs needs to show the table of strategies on newsprint (such as the one in the box below).
- Add any strategies that have been developed from the meeting and remove any strategies that are not considered suitable.
- Reach a consensus on the strategies.
- Develop a plan for each strategy. When will the strategy be implemented? Who will be responsible for each strategy?

Examples of implementation plan

Possible problem	Realistic Strategies	When the strategy will be implemented	Person responsible
Home delivery	Women's group teaches other women about the danger of home delivery	June 2017	Women Group Members
	Women's group organizes street drama	July 2017	Women Group Members
	Women groups and male champions visit couples at home to discuss birth plans	September 2017	Women Group Members and Male champions
Early ANC attendance	Women's group teaches other women about the importance of early ANC attendance	June 2017	Women Group Members
	Male champions teaches other men the importance of early ANC attendance	July 2017	Men Group Members
Home delivery	Women's group teaches other women about the danger of home delivery	June 2017	Women Group Members
	Women's group organizes street drama	July 2017	Women Group Members

Meeting 8: Evaluating together

The Women's Group Facilitators will guide the women and communities in their area through a process of evaluating the strategies they have implemented and planning for the future in terms of improving the strategies and identifying new problems and how to address them.

For peer review only - http://bmjopen.bmj.com/site/about/guidelines.xhtml
